# Comprehensive behavioral phenotyping of male *Sulf1*-deficient mice reveals reduced locomotor activity, altered forelimb gait, and reduced prepulse inhibition

**DOI:** 10.1186/s13041-026-01353-8

**Published:** 2026-10-17

**Authors:** Kazuko Keino-Masu, Markos Michail Chatzigiannis, Hirotaka Shoji, Nobuyuki Yamasaki, Takuya Okada, Yasue Horiuchi, Tadao Arinami, Yoko Hagino, Kazutaka Ikeda, Tsuyoshi Miyakawa, Masayuki Masu

**Affiliations:** 1 Department of Molecular Neurobiology, Institute of Medicine, University of Tsukuba, 1-1-1 Tennodai, Tsukuba, 305-8575 Ibaraki Japan; 2 Graduate School of Comprehensive Human Sciences, University of Tsukuba, Tsukuba, Ibaraki 305-8575 Japan; 3 Division of Systems Medical Science, Center for Medical Science, Fujita Health University, Toyoake, Aichi 470-1192 Japan; 4 Genetic Engineering and Functional Genomics Group, Horizontal Medical Research Organization (HMRO), Kyoto University Graduate School of Medicine, Sakyo-ku, Kyoto, 606-8501 Japan; 5 Department of Molecular Genetics, Institute of Basic Medical Sciences, University of Tsukuba, Ibaraki, 305-8575 Japan; 6 CREST, Japan Science and Technology Agency, Kawaguchi, Saitama 332-0012 Japan; 7 Addictive Substance Project, Tokyo Metropolitan Institute of Medical Science, Setagaya-ku, Tokyo, 156-8506 Japan; 8 Shizuoka Graduate University of Public Health, Shizuoka, Shizuoka 420-0881 Japan; 9 Department of Neuropsychopharmacology, National Institute of Mental Health, National Center of Neurology and Psychiatry, Kodaira, Tokyo, 187-8553 Japan

**Keywords:** Behavioral test battery, Gait analysis, Knockout mouse, Open field test, Prepulse inhibition, Sulfatase 1 (Sulf1)

## Abstract

Sulfatase 1 (Sulf1) is an extracellular sulfatase that regulates multiple signaling pathways by removing 6-*O*-sulfate from heparan sulfate. Although *Sulf1* has established roles in development and cancer, its functions in the adult brain remain incompletely characterized. Recently we reported that *Sulf1* is expressed in the nucleus accumbens, overlapping with dopamine D1 and D2 receptors, and that *Sulf1* knockout (KO) mice showed altered reward and aversion learning. However, given that *Sulf1* is expressed in many other brain regions, including the prefrontal cortex, midline thalamic nuclei, etc., *Sulf1* may have unknown brain functions. To explore the *Sulf1* functions across a broad range of contexts, we here perform a comprehensive behavioral analysis of male *Sulf1* KO mice, together with a re-analysis of the original data in which the false discovery rate (FDR) was controlled within defined groups of tests. *Sulf1* KO mice traveled less than wild-type (WT) mice in the open field test, and this reduction was reproduced in an independent cohort. Gait analysis on a treadmill revealed a redistribution of the forelimb stance phase, with a larger proportion spent braking and a smaller proportion spent propelling, together with an increased absolute paw angle; these forelimb changes were reproduced in 2 independent cohorts and at 2 treadmill speeds. Distance traveled was also reduced in a three-chamber apparatus and, before drug injection, at nominal significance in the home cage. *Sulf1* KO mice showed larger acoustic startle responses and reduced prepulse inhibition (PPI); the reduction in PPI was observed in all 5 drug-free or saline-treated sessions conducted in 2 independent cohorts and was also observed after haloperidol (0.3 or 1 mg/kg) and, at nominal significance, after clozapine (3 mg/kg) treatment. The locomotor, vertical, and stereotypic responses to methamphetamine were attenuated in *Sulf1* KO mice, with nominally significant drug × genotype interactions. Increased baseline-subtracted contextual freezing provides a promising lead for further confirmation. Taken together, adult male *Sulf1* KO mice exhibit alterations in locomotor output, forelimb gait, and sensorimotor gating, implicating that *Sulf1* deficiency leads to a wide range of neurobehavioral abnormalities.

**Supplementary Information:**

The online version contains supplementary material available at https://doi.org/10.1186/s13041-026-01353-8.

## Introduction

Heparan sulfate (HS) is a linear polysaccharide attached to core proteins at the cell surface and in the extracellular matrix, where its sulfation pattern determines which growth factors, morphogens and guidance molecules it binds [1–3]. Among the enzymes that shape this pattern, the extracellular endosulfatases Sulf1 and Sulf2 act after HS has been synthesized, selectively removing 6-*O*-sulfate groups and thereby switching the signaling output of HS chains [1–3]. Sulf1 and Sulf2 modulate GDNF, Wnt, FGF and Shh signaling, and are required for esophageal innervation, neonatal survival, and formation of the corticospinal tract [4–6].

In the adult brain, *Sulf1* is expressed in regions relevant to reward processing, and is co-expressed with dopamine D1 and D2 receptors in the nucleus accumbens [7]. Consistent with this expression pattern, *Sulf1* KO mice show altered reward and aversion learning through distinct D1 and D2 receptor pathways [8]. An earlier study reported reduced hippocampal spine density and impaired long-term potentiation in *Sulf1*-deficient mice [9]. Whether these molecular and synaptic changes translate into a coherent behavioral phenotype has not been established.

We therefore subjected male *Sulf1* KO mice to a comprehensive behavioral test battery [10, 11] covering general health, motor function, gait, spontaneous activity, anxiety- and depression-related behavior, sensorimotor gating, learning and memory, and social behavior, and complemented the battery with pharmacological challenges and in vivo microdialysis. Because a battery of this size generates a large number of dependent measures, we re-analyzed the original data and controlled the false discovery rate within defined groups of tests [12], so that the strength of evidence for each finding can be judged explicitly. We also examined whether variation in the human *SULF1* gene is associated with psychiatric disorders in which deficits in prepulse inhibition (PPI) have been reported, and evaluated the resulting associations against current large-scale genome-wide association study (GWAS) data [13, 14].

## Materials and methods

Full details of the animals, behavioral procedures, pharmacological challenges, in vivo microdialysis, and the human genetic association study are provided in Supplementary Materials 3. Only the points required to interpret the results are summarized here.

### Animals

*Sulf1* KO mice were generated by homologous recombination in embryonic stem cells and backcrossed to the C57BL/6 N background for 10 generations [15]. Only male mice were examined. Three independent cohorts were used, comprising 73 WT and 73 KO mice in total. Behavioral testing was conducted as described previously [10, 11].

### Pharmacological challenges

In the open field test, methamphetamine (3 mg/kg) or saline was administered intraperitoneally 60 min after the mice entered the arena, and behavior was recorded for a further 120 min; methamphetamine- and saline-treated groups were run in parallel in the same session. In the startle and PPI test, haloperidol (0.3 or 1 mg/kg) or clozapine (3 mg/kg) was administered intraperitoneally before the start of the session, and a saline-treated group was run in parallel in each drug session. Three drug sessions were conducted in the same cohort, in the order haloperidol 0.3 mg/kg, clozapine, haloperidol 1 mg/kg. Each session included WT–saline, KO–saline, WT–drug, and KO–drug groups (*n* = 15 per group), with treatment assignments changing across sessions.

### Statistical analysis

Data were analyzed with a two-tailed *t*-test, two-way ANOVA, two-way repeated measures ANOVA, or two-way mixed model ANOVA. For repeated-measures data, a single *p* value for the genotype main effect was used and counted as one test. Because a large number of measures was examined, the false discovery rate (FDR) was controlled at 5% within each group of tests (“test family”) by the Benjamini–Hochberg procedure [12]. The 57 drug-free genotype comparisons listed in Supplementary Table S1a were treated as one family of tests and those obtained from the hypothesis-driven follow-up experiments (methamphetamine, haloperidol and clozapine challenges with their parallel saline arms, and in vivo microdialysis) as a second family (30 measures). The two Day-1 baseline comparisons in fear conditioning and the 4 delayed-trial comparisons in the eight-arm radial maze were not included in Family 1 and are reported with unadjusted *p* values. The measures obtained from the home-cage social interaction test, including those computed separately for the light and the dark phase, and the pre-injection measures of the home-cage drug challenges, belong to the first family; the post-injection measures of those challenges belong to the second family. Gait analysis, which was performed on a separate apparatus in different cohorts and yields a large set of internally redundant measures, was treated as a third family (171 comparisons). Whether the genotype effect differed between the forelimb and the hindlimb was tested directly. Because the 2 limb pairs were recorded from the same animals in the same run, a within-animal difference score, *D* = forelimb value − hindlimb value, was computed for each mouse and compared between genotypes by a two-tailed *t*-test; this is equivalent to the genotype × limb interaction term of a mixed-model analysis of variance with limb as the within-subject factor. The test was applied to the 2 measures that index distinct aspects of the phenotype, %BrakeStance (contact timing) and absolute paw angle (paw orientation), in each cohort and at each belt speed. Because these measures are bounded percentages and strictly positive angles respectively, the interaction was repeated after logit and log transformation as a sensitivity analysis (Supplementary Materials 1). A measure with an adjusted *p* value (*q*) below 0.05 within its family is described as significant after within-family false discovery rate (FDR) correction; a measure significant only before adjustment is described as nominal. For the fear conditioning test, 2 baseline-normalized indices were computed in addition to conventional percentage freezing, following Sato et al. [16]: freezing subtraction, defined as freezing during the test session minus freezing during the Day-1 baseline period (min 1–2, before the first foot shock), and the activity suppression ratio, defined as the distance traveled during the test divided by the sum of the distances traveled during the Day-1 baseline and the test. All comparisons included in Families 1 and 2, with raw and adjusted *p* values, are listed in Supplementary Table S1 (Supplementary Materials 4). The complete results for the gait family, with group means, effect estimates and 95% confidence intervals, are provided in Supplementary Materials 1. Exploratory drug × genotype interactions were tested separately for each antipsychotic session by two-way ANOVA of per-animal means (startle amplitude averaged across stimulus intensities; PPI averaged across prepulse levels for each startle intensity). The 9 interaction tests were adjusted as a separate family; effect estimates and 95% confidence intervals are reported in Supplementary Table S2.

## Results

### General health and motor function in *Sulf1* KO mice

**Fig. 1:**
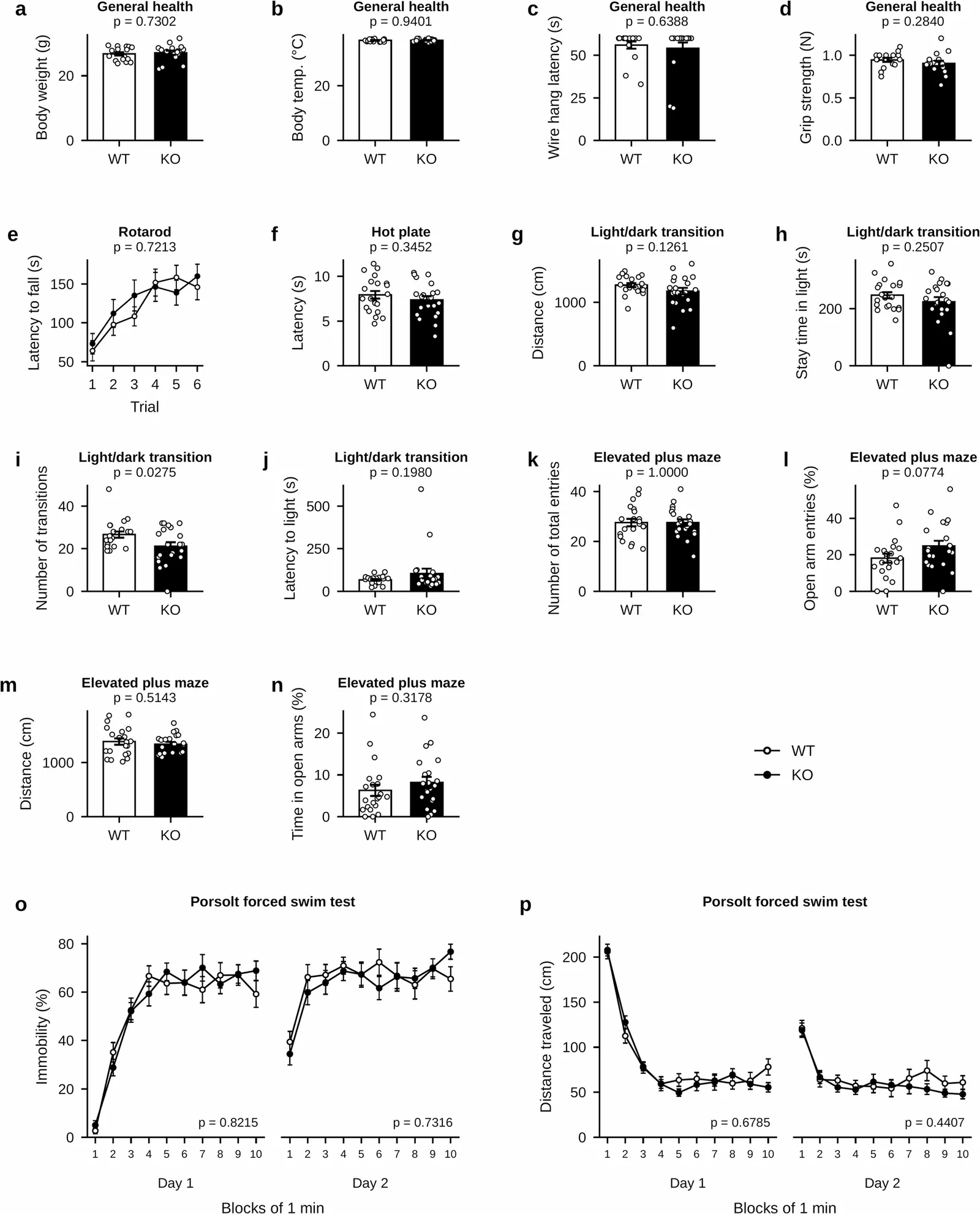
General health, motor function, anxiety- and depression-related behavior. (**a–d**) Body weight, body temperature, wire hang latency and grip strength. (**e**) Rotarod test, latency to fall across 6 trials. (**f**) Hot plate test. (**g–j**) Light/dark transition test. (**k–n**) Elevated plus maze. (**o**) Porsolt forced swim test, percentage immobility in blocks of 1 min on days 1 and 2. (**p**) Distance traveled in the same test.　Bars show the mean ± SEM and symbols show individual mice. *p* values are for the genotype main effect

Body weight, body temperature, wire hang latency, and grip strength did not differ between genotypes (Fig. 1a–d; *p* = 0.73, 0.94, 0.64 and 0.28, respectively), and no gross health abnormality was noted. Motor coordination and balance assessed by the rotarod test, and nociceptive threshold assessed by the hot plate test, also showed no significant genotype differences (Fig. 1e, f; *p* = 0.72 and 0.35). These assays did not detect a gross motor deficit.

### Spontaneous locomotor activity is reduced in the open field test

**Fig. 2:**
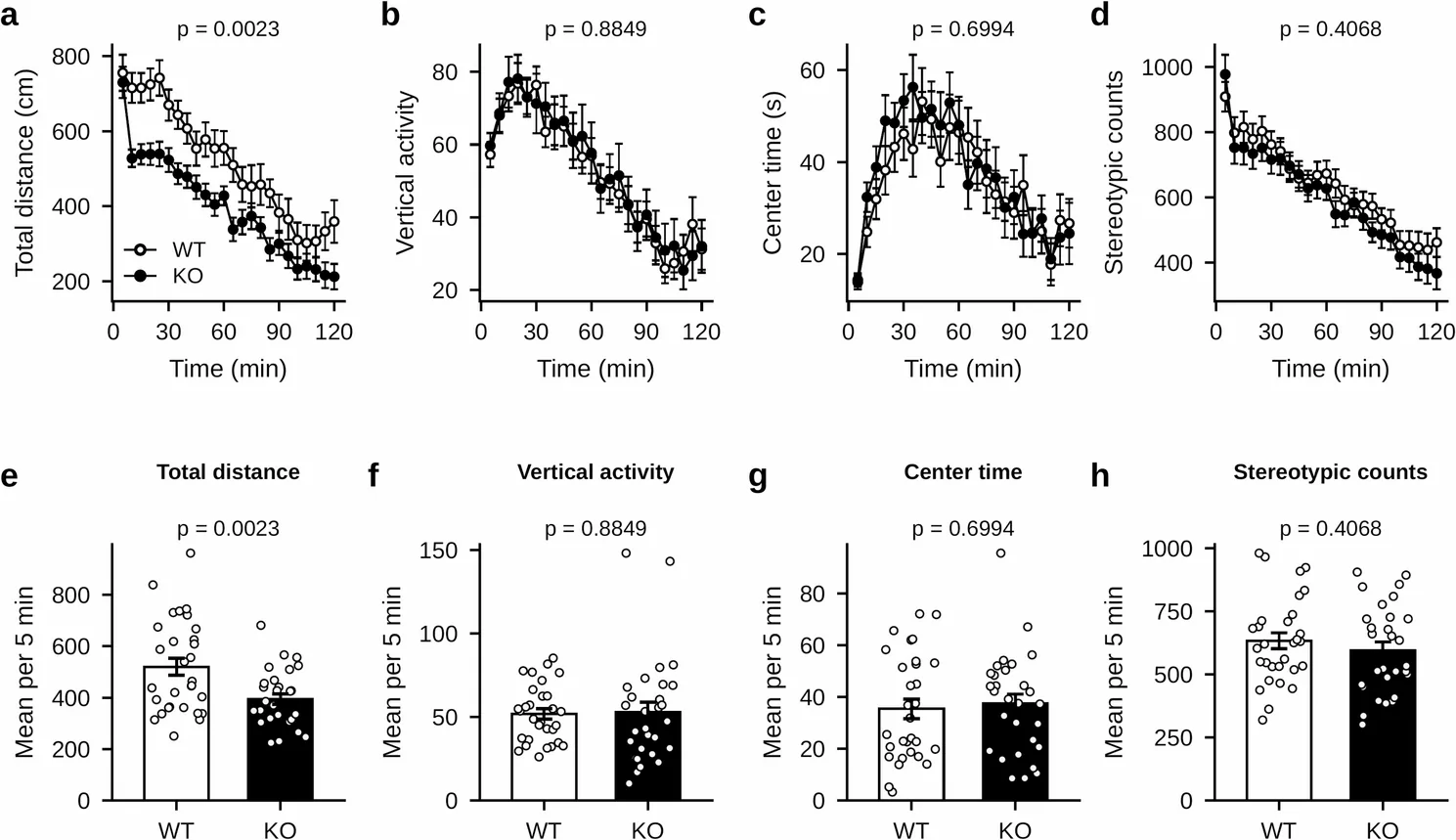
Open field test. (**a–d**) Time course of total distance traveled, vertical activity, time spent in the center area, and stereotypic counts, in 5-min bins over 120 min. (**e–h**) Corresponding per-animal means; bars show the mean ± SEM and symbols show individual mice. Open symbols and bars, WT (*n* = 30); filled symbols and bars, KO (*n* = 29). Bars show the mean ± SEM and symbols show individual mice. *p* values are for the genotype main effect

In the open field test, total distance traveled was significantly lower in *Sulf1* KO mice than in WT mice (Fig. 2a, e; *p* = 0.0023, *q* = 0.044, significant after within-family FDR correction), whereas vertical activity, time spent in the center area, and stereotypic counts did not differ (Fig. 2b–d, f–h; *p* = 0.88, 0.70 and 0.41). The difference was present from the first time bin and the 2 genotypes habituated in parallel, indicating a lower overall level of activity rather than an altered rate of habituation. The same result was obtained in an independent cohort (*n* = 21 per genotype; *p* = 0.0007; Supplementary Materials 4, 7 replication across cohorts), confirming the reproducibility of this finding. Reduced locomotion was also apparent in 2 further settings: distance traveled in a three-chamber apparatus was lower in *Sulf1* KO mice (Section "Anxiety-, depression-related behavior, learning, memory and social behavior" ; *p* = 0.0013, *q* = 0.037, significant after within-family FDR correction), as was home-cage activity during the 60 min preceding drug injection (Section "Home-cage activity and the response to MK-801"; *p* = 0.034, *q* = 0.244; nominal). The reduction in locomotor activity is therefore observed across 3 different apparatus and in multiple test sessions.

### Forelimb gait is altered in *Sulf1* KO mice

**Fig. 3:**
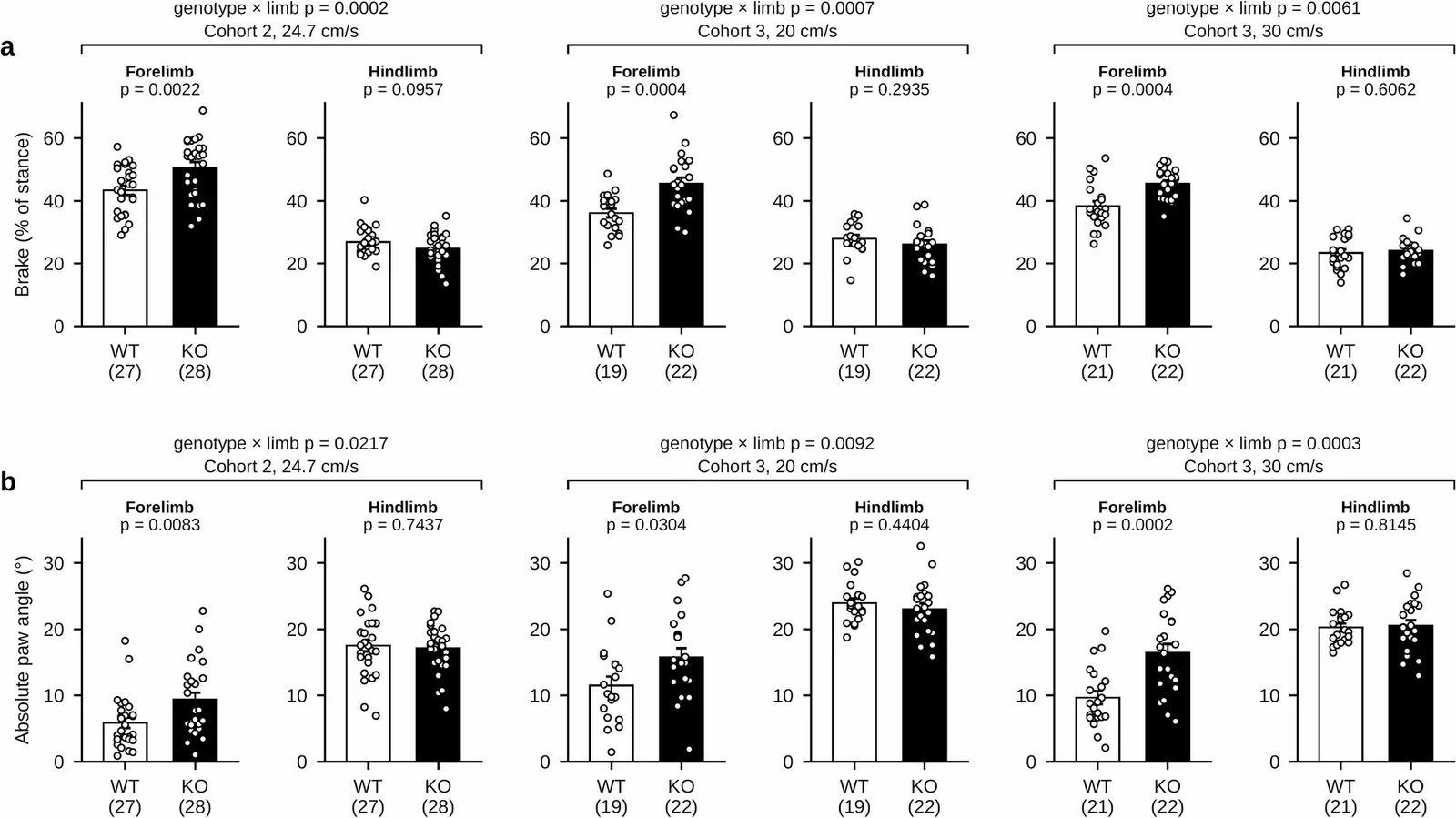
Gait analysis on a treadmill. (**a**) Proportion of the stance phase spent braking (%BrakeStance). (**b**) Absolute paw angle. Each pair of panels shows the forelimb and the hindlimb of the same animals in one condition: cohort 2 at a belt speed of 24.7 cm/s, and cohort 3 at 20 and at 30 cm/s. Bars show the mean ± SEM, symbols show individual mice, and the number of mice per group is give*n* below each bar. Open bars and open symbols, WT; filled bars and filled symbols, *Sulf1* KO. The *p* value above each panel is an unadjusted two-tailed Student *t*-test of the genotype effect; the value above each bracket is the genotype × limb interaction, tested on the within-animal difference score. Because the braking and propulsion phases partition the stance phase, %PropelStance = 100 − %BrakeStance by definition; the propulsion measures and all remaining gait variables, with raw and Benjamini–Hochberg adjusted values for all 171 comparisons, are given in Supplementary Materials 1. Bars show the mean ± SEM and symbols show individual mice

Because locomotor activity was reduced while no gross motor deficit was detected, we examined gait on a treadmill. In the forelimb, the proportion of the stance phase spent braking was increased and the proportion of the stride spent propelling was decreased in *Sulf1* KO mice, and the absolute paw angle was increased (Fig. 3a, b; the propulsion measures are given in Supplementary Materials 1). These changes were observed in 2 independent cohorts (cohorts 2 and 3) and, in cohort 3, at both treadmill speeds tested (braking, *p* = 0.0022, 0.00035 and 0.00041; propulsion, *p* = 0.00073, 0.00089 and 0.00014; paw angle, *p* = 0.0083, 0.030 and 0.00020, for cohort 2 and for cohort 3 at 20 and 30 cm/s, respectively). Sixteen of the 171 comparisons in the gait family passed the false discovery rate threshold, the smallest adjusted value being *q* = 0.010; all 16 were forelimb comparisons. Because several DigiGait variables share component durations and denominators — %BrakeStance and %PropelStance, for example, sum to 100 by definition — these entries are correlated readouts of the same underlying change rather than 16 independent abnormalities.

Among the hindlimb comparisons, only the proportion of the stride spent propelling reached nominal significance, and only in cohort 2 (*p* = 0.016), with no significant difference at either treadmill speed in cohort 3 (*p* = 0.40 and 0.57). Neither stride length nor stride frequency differed between genotypes in the forelimb in any condition, and the 95% confidence interval of the genotype difference included zero throughout (stride length, *p* = 0.26, 0.53 and 0.25; stride frequency, *p* = 0.26, 0.61 and 0.34), so the forelimb contact-timing phenotype was not accompanied by a detectable genotype difference in forelimb stride length or stride frequency. No hindlimb comparison survived correction for multiple comparisons. The difference between the limbs was confirmed by a direct test. Using the within-animal difference score *D* = forelimb − hindlimb, the genotype × limb interaction was significant in every cohort and at every belt speed, for both the timing measure (%BrakeStance: *p* = 0.0002, 0.0007 and 0.0061) and the orientation measure (absolute paw angle: *p* = 0.022, 0.0092 and 0.0003, for cohort 2 and for cohort 3 at 20 and 30 cm/s, respectively; Fig. 3). The same 6 interactions remained significant after logit or log transformation. In each of the 6 conditions the 95% confidence interval of the genotype effect excluded zero for the forelimb and included zero for the hindlimb (Supplementary Materials 1). The gait phenotype of *Sulf1* KO mice is therefore selective for the forelimb.

### The behavioral response to methamphetamine is attenuated

**Fig. 4:**
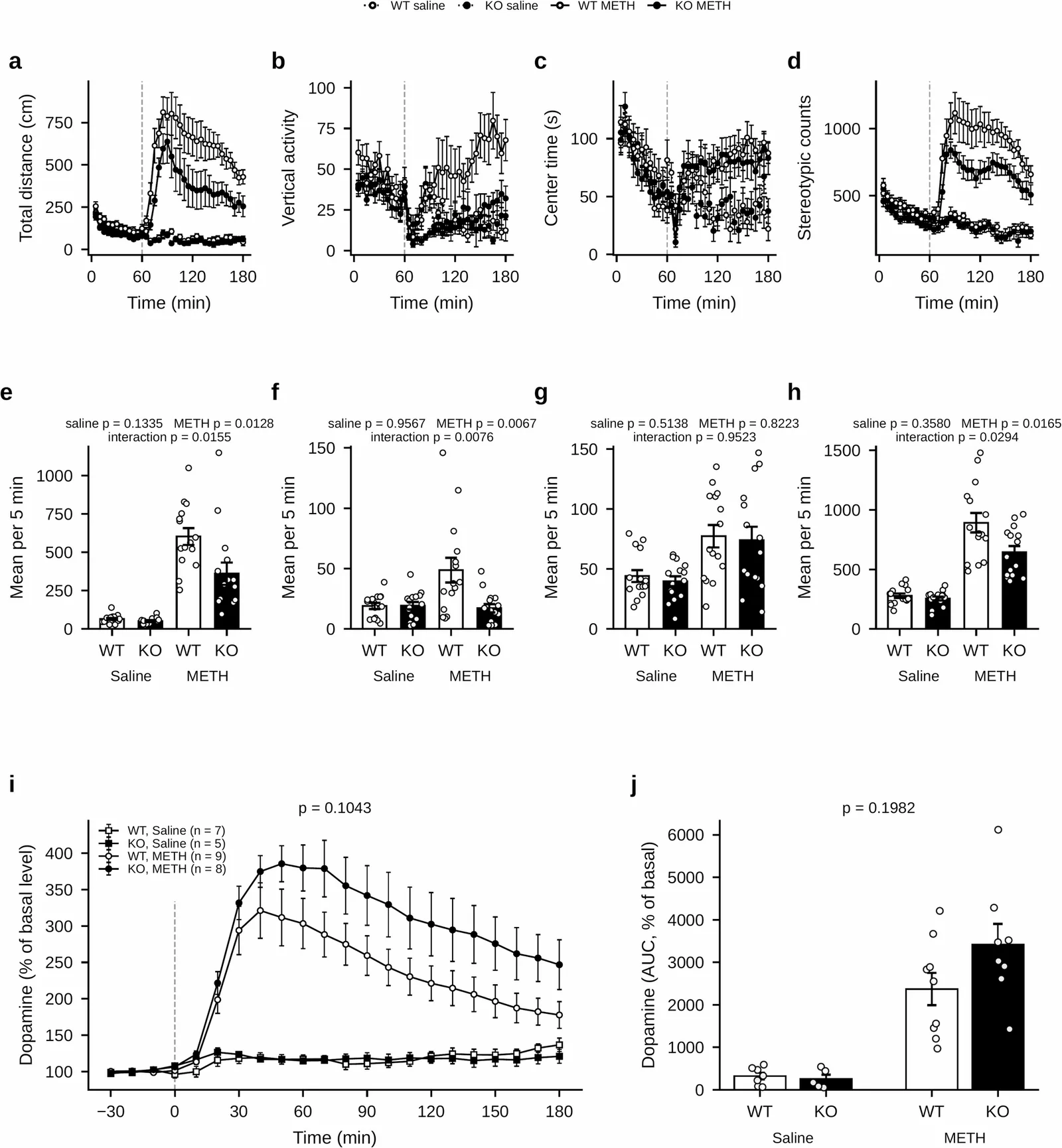
Methamphetamine challenge and accumbal dopamine release. **(a–d**) Time course of open field measures over 180 min; methamphetamine (METH, 3 mg/kg) or saline was intraperitoneally administered at 60 min (dashed line). (**e–h**) Per-animal means over the post-injection period (60–180 min), with *p* values for the genotype effect within each treatment and for the drug × genotype interaction. *n* = 15 per genotype and treatment. (**i, j**) Extracellular dopamine in the nucleus accumbens, expressed as a percentage of the basal level and as the area under the curve. At time 0 (dashed line), methamphetamine (METH, 1 mg/kg) or saline was subcutaneously administered. Bars show the mean ± SEM and symbols show individual mice

Methamphetamine (3 mg/kg) increased total distance traveled, vertical activity, and stereotypic counts in both genotypes, but the responses of *Sulf1* KO mice were smaller than those of WT mice (Fig. 4a–h; post-injection *p* = 0.013, 0.0067 and 0.017; *q* = 0.044, 0.044 and 0.049, all significant after within-family FDR correction). A saline-treated group run in parallel showed no genotype difference in vertical activity or stereotypic counts (*p* = 0.96 and 0.36), and the drug × genotype interactions were nominally significant for total distance traveled, vertical activity, and stereotypic counts (*p* = 0.016, 0.0076 and 0.029) but not for time spent in the center area (*p* = 0.95). These interactions support attenuation of the methamphetamine response beyond the lower baseline activity of the KO mice.

To determine whether the attenuated response reflected reduced dopamine release, we measured extracellular dopamine in the nucleus accumbens by in vivo microdialysis [17]. Methamphetamine produced a marked increase in both genotypes, and neither the time course nor the area under the curve differed significantly between genotypes; if anything, values were higher in *Sulf1* KO mice (Fig. 4i, j; *p* = 0.10 and 0.20; *q* = 0.196 and 0.285). Extracellular serotonin, measured in the same samples, showed a similar pattern (*p* = 0.13 and 0.45; per-animal values in Supplementary Materials 2). Serotonin was an exploratory measure and was not included in the multiplicity correction. The attenuated behavioral response was not accompanied by a detectable reduction in dopamine release, suggesting altered responsiveness downstream of dopamine release or at the circuit level.

### Startle responses are increased and prepulse inhibition is reduced

**Fig. 5:**
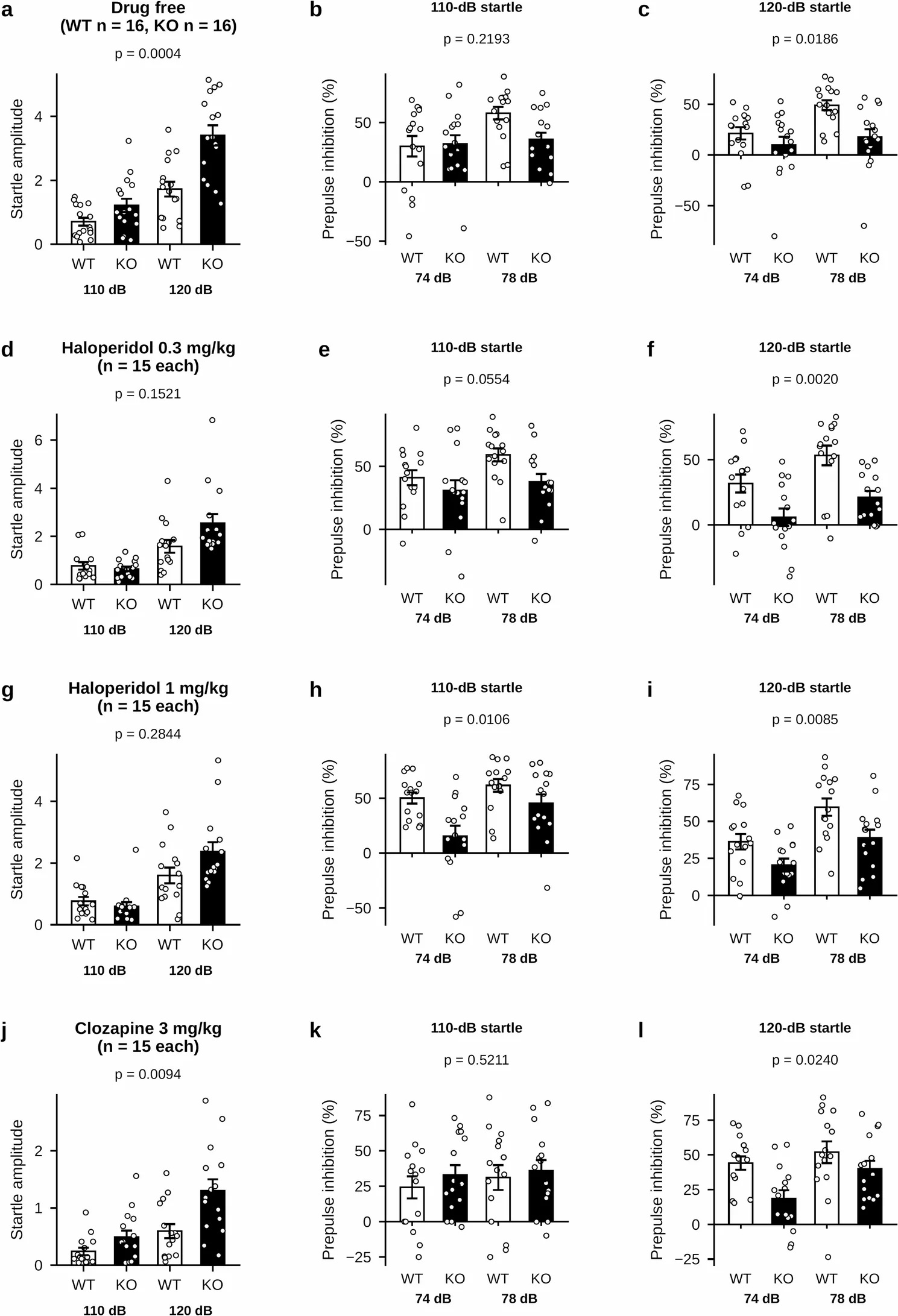
Acoustic startle response and prepulse inhibition. (**a–c**) Drug-free session (WT *n* = 16, KO *n* = 16). (**d–f**) After haloperidol 0.3 mg/kg. (**g–i**) After haloperidol 1 mg/kg. (**j–l**) After clozapine 3 mg/kg (*n* = 15 per genotype in each drug-treated group). Parallel saline-treated WT and KO groups (*n* = 15 per genotype) were included in each session; their results and the exploratory interactions are reported in Supplementary Material 4, TablesS1 and S2. In each row, the left panel shows startle amplitude at 110 and 120 dB, and the middle and right panels show prepulse inhibition at the 110-dB and 120-dB startle stimuli, each at prepulse levels of 74 and 78 dB. Bars show the mean ± SEM and symbols show individual mice. The *p* values are for the genotype main effect across prepulse levels

*Sulf1* KO mice showed significantly larger acoustic startle responses to 110- and 120-dB stimuli than WT mice (Fig. 5a; *p* = 0.0004, *q* = 0.023, significant after within-family FDR correction). PPI was lower in *Sulf1* KO mice under the 120-dB startle condition (Fig. 5c; *p* = 0.019) but not under the 110-dB condition (Fig. 5b; *p* = 0.22). Within the drug-free family the reduction in PPI was nominal (*q* = 0.212); however, it was reproduced in every session in which it was measured, comprising 2 independent cohorts and 5 drug-free or saline-treated sessions (*p* = 0.0035, 0.019, 0.013, 0.048, and 0.0012), with a consistent direction and effect size (Cohen’s *d* = 0.76–1.32).

Because PPI is expressed as a proportion of the startle response, a larger startle response can in principle produce a lower value of PPI in the absence of a true gating deficit. Two drug-treated comparisons address this possibility. First, after administration of haloperidol at 1 mg/kg (a dopamine D2 receptor antagonist) the genotype difference in startle amplitude was no longer statistically significant (Fig. 5g; *p* = 0.28), whereas the reduction in PPI persisted and was in fact significant under both the 110- and the 120-dB startle conditions (Fig. 5h, i; *p* = 0.011 and 0.0085; *q* = 0.044 for both). Second, after administration of clozapine the reduction in PPI at the 120-dB condition was still present at nominal significance (Fig. 5l; *p* = 0.024, *q* = 0.060), although the genotype difference in startle amplitude also remained (Fig. 5j; *p* = 0.0094). Exploratory drug × genotype interactions with the parallel saline groups were not significant (all *p* ≥ 0.198, *q* ≥ 0.768; Supplementary Table S2). These results do not establish pharmacological non-responsiveness or exclude a contribution of startle amplitude to the PPI difference.

### Anxiety-, depression-related behavior, learning, memory and social behavior

**Fig. 6:**
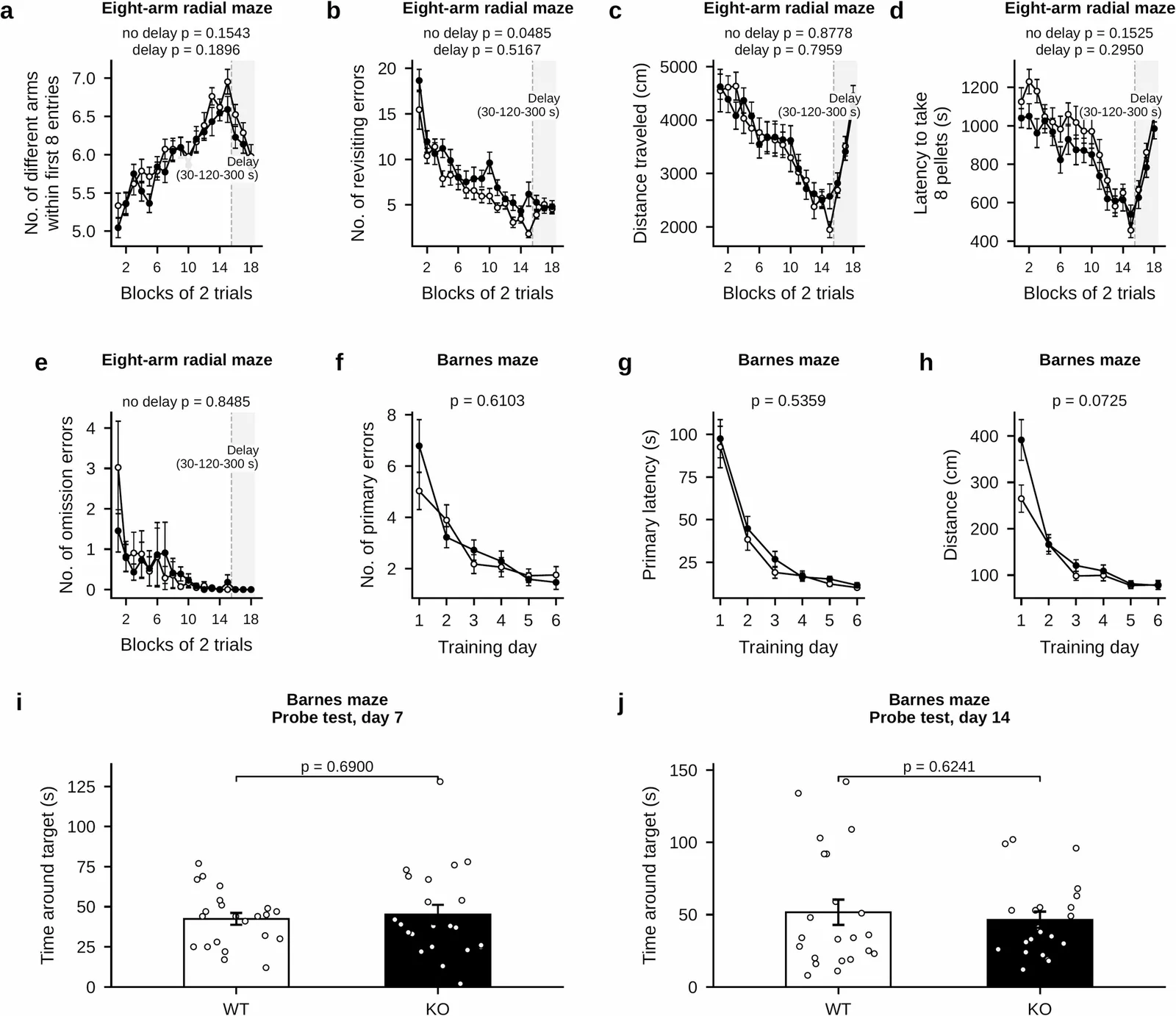
Spatial working and reference memory. (**a–e**) Eight-arm radial maze, plotted in blocks of 2 trials: number of different arms chosen within the first 8 entries, number of revisiting errors, distance traveled, latency to take all 8 pellets, and number of omission errors. The horizontal bar marks the final trials, in which delays of 30, 120 and 300 s were imposed after the fourth pellet had been taken; *p* values are given separately for the trials without and with delay. (**f–h**) Barnes maze: number of primary errors, primary latency, and distance traveled across training days. (**i, j**) Barnes maze probe tests on days 7 and 14. Bars show the mean ± SEM and symbols show individual mice

**Fig. 7:**
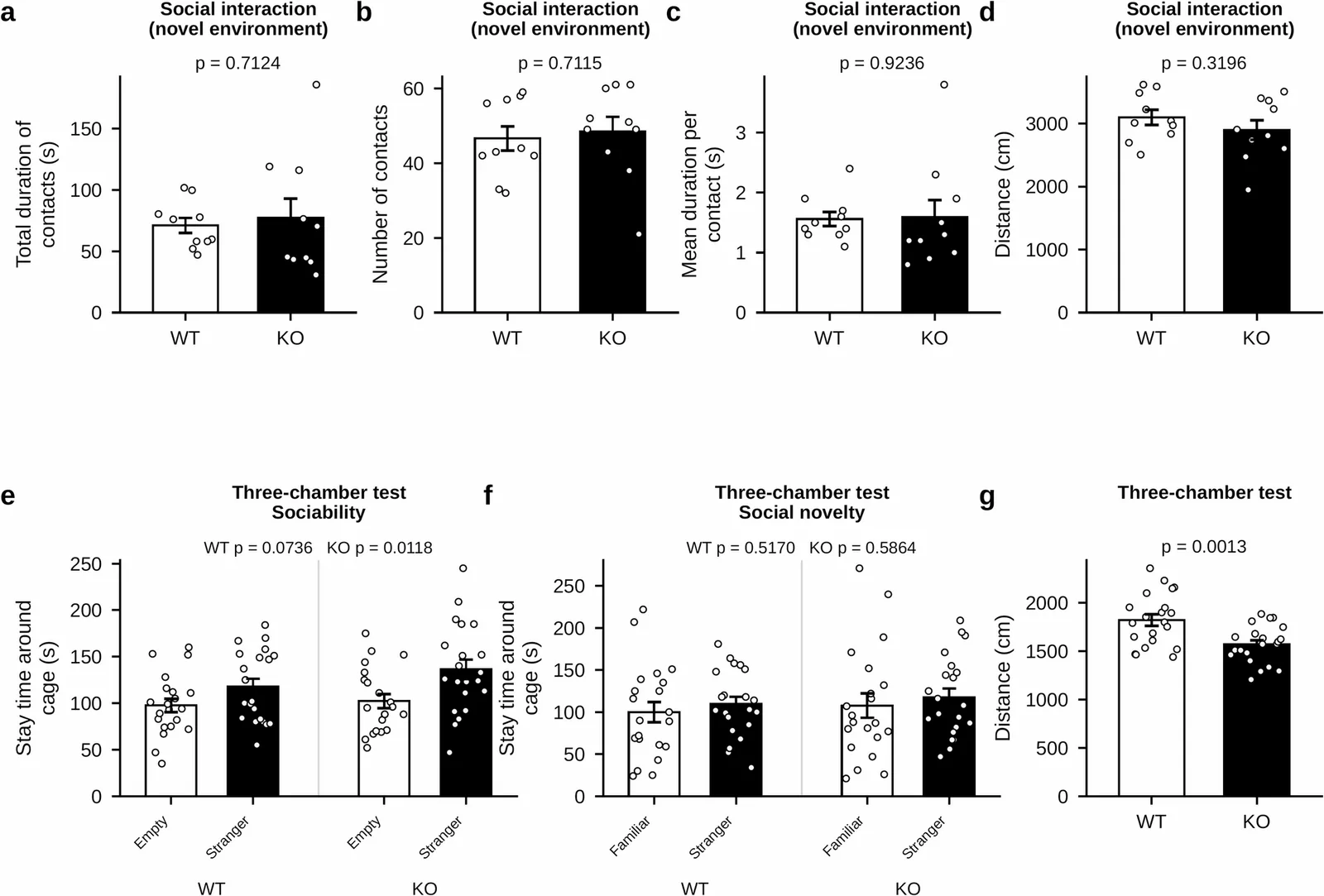
Social behavior. (**a–d**) Social interaction test in a novel environment. (**e**) Three-chamber test, sociability phase: stay time around the empty cage and around the cage containing a stranger mouse. (**f**) Three-chamber test, social novelty phase. (**g**) Distance traveled in the three-chamber apparatus. Bars show the mean ± SEM and symbols show individual mice

**Fig. 8:**
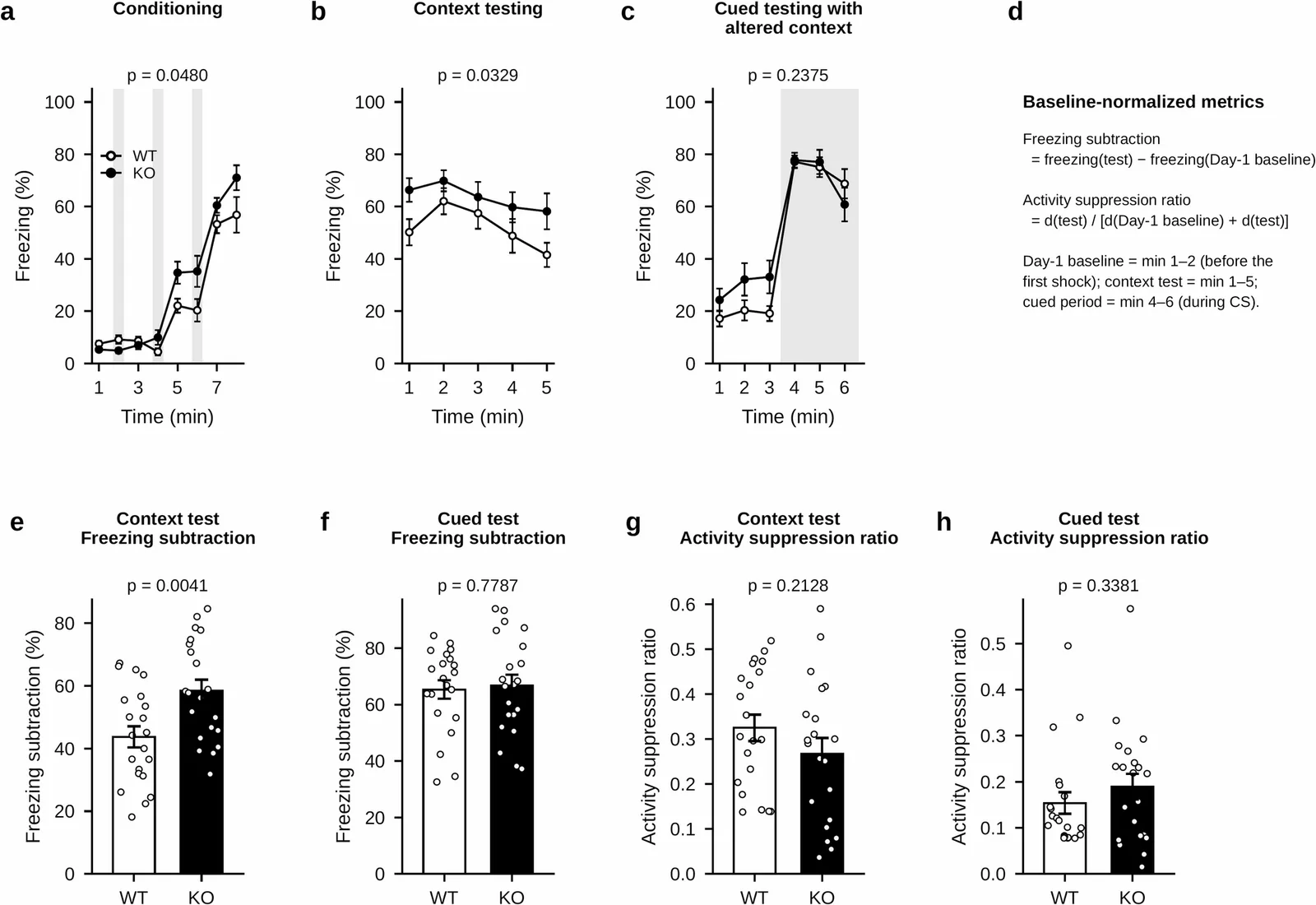
Contextual and cued fear conditioning. (**a**) Freezing during the conditioning session; shaded bars indicate the tone–shock pairings. (**b**) Freezing during the context test. (**c**) Freezing during the cued test in an altered context; the shaded area indicates presentation of the conditioned stimuli. (**d**) Definitions of the 2 baseline-normalized indices. (**e**, **f**) Freezing subtraction for the context and cued tests. (**g, h**) Activity suppression ratio for the context and cued tests. Bars show the mean ± SEM and symbols show individual mice

No genotype differences that survived correction for multiple comparisons were observed in the light/dark transition test, the elevated plus maze, or the Porsolt forced swim test (Fig. 1g–p). The number of transitions in the light/dark transition test differed nominally (*p* = 0.028) but not after correction (*q* = 0.244). Spatial working-memory measures in the eight-arm radial maze and spatial-reference memory measures in the Barnes maze also showed no significant genotype differences after correction (Fig. 6a–j). In the eight-arm radial maze, trials 1–29 were conducted without a delay and the last 6 trials with delays of 30, 120 and 300 s imposed after the fourth pellet had been taken. During the trials without delay, the number of revisiting errors was nominally higher in *Sulf1* KO mice (*p* = 0.049, *q* = 0.248), whereas the number of different arms chosen within the first 8 entries, a measure of working memory that is relatively independent of locomotor activity, did not differ (*p* = 0.15); distance traveled, latency to collect all 8 pellets, and omission errors also showed no significant genotype differences (*p* = 0.88, 0.15 and 0.85). During the trials with delay, no measure differed between genotypes in unadjusted analyses (*p* = 0.19, 0.52, 0.80 and 0.30 for different arm choices, revisiting errors, distance traveled, and latency, respectively). In the three-chamber test, both genotypes spent more time near the cage containing a stranger mouse than near the empty cage, with nominal significance only in *Sulf1* KO mice (WT, *p* = 0.074; KO, *p* = 0.012); neither genotype showed a significant preference for the novel over the familiar mouse in the second phase (Fig. 7e, f). Distance traveled in the three-chamber apparatus was lower in *Sulf1* KO mice (Fig. 7g). No significant genotype difference in social interaction in a novel environment was detected (Fig. 7a–d). In contextual and cued fear conditioning, freezing during conditioning and during the context test differed nominally between genotypes (*p* = 0.048 and 0.033) but neither difference survived correction (*q* = 0.248 and 0.244; Fig. 8a, b).

### Contextual fear is nominally increased when normalized to baseline

Because percentage freezing conflates memory with baseline locomotor and anxiety-related traits [16], we also computed 2 baseline-normalized indices. *Sulf1* KO mice froze less than WT mice during the Day-1 baseline period, before any foot shock had been delivered (8.3% versus 5.1%; *p* = 0.0087, unadjusted), whereas distance traveled during that period did not differ (*p* = 0.53, unadjusted). After subtracting this baseline, freezing during the context test was higher in *Sulf1* KO mice than in WT mice, and the difference was larger than that obtained with conventional freezing (freezing subtraction, 43.7% versus 58.4%; *p* = 0.0041, *q* = 0.058; Cohen’s *d* = 0.94; Fig. 8e). The corresponding index for the cued test did not differ between genotypes (*p* = 0.78; Fig. 8f). The activity suppression ratio was in the same direction for the context test but did not reach significance (0.325 versus 0.268; *p* = 0.21; Fig. 8g), and did not differ for the cued test (*p* = 0.34; Fig. 8h).

This is the only fear-conditioning measure that approaches the within-family FDR threshold, and it suggests enhanced rather than impaired contextual fear. The adjusted *p* value nevertheless lies above 0.05 and is sensitive to the number of measures included in the family. Because the 2 normalized indices differ in statistical support, and because one is based on freezing and the other on locomotor activity, this finding should be regarded as promising but in need of replication.

### Social interaction in the home cage

**Fig. 9:**
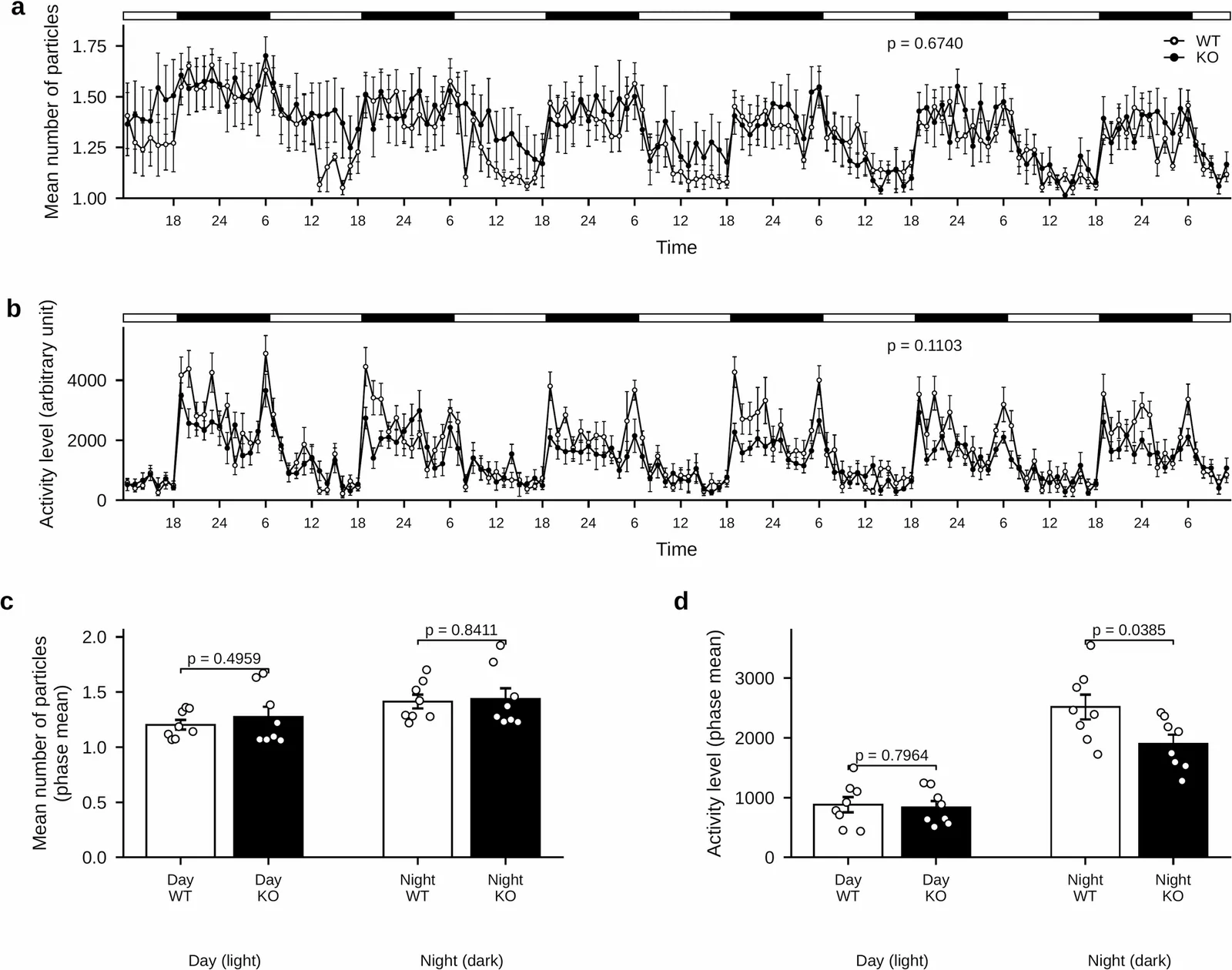
Social interaction in the home cage. *Sulf1* KO and WT mice were housed in same-genotype pairs and monitored over 7 days; the first 24 h were discarded as habituation (WT, *n* = 8 cage pairs; KO, *n* = 8 cage pairs). The cage pair is the unit of analysis. Open circles (**a, b**) and open bars (**c, d**), WT; filled circles (**a, b**) and filled bars (c, d), *Sulf1* KO. Horizontal bars above (**a**) and (**b**) indicate the light (open) and dark (filled) phases; lights were off from 19:00 to 07:00, and each hourly bin is labeled by its start time. (**a**) Hourly mean number of particles, an index of social contact: 1 particle indicates that the 2 mice were in contact and 2 particles that they were apart. (**b**) Hourly home-cage activity level in arbitrary units, measured as the number of pixels changed between successive frames. (**c**) Mean number of particles during the light and the dark phase. (**d**) Home-cage activity during the light and the dark phase. Circles (**a, b**) and bars (**c, d**) show the mean ± SEM; *p* values are for the genotype main effect

Social behavior was also assessed under undisturbed conditions by housing same-genotype pairs together and monitoring them over 7 days. Social contact was indexed by the mean number of particles detected by image analysis, 1 particle indicating that the 2 mice were in contact and 2 that they were apart, and locomotor activity was measured in parallel as the number of pixels changed between successive frames [18]. Eight cage pairs per genotype were recorded, and the cage pair was treated as the unit of analysis; the first 24 h were discarded as a habituation period. The mean number of particles did not differ between genotypes (Fig. 9a; *p* = 0.67), and home-cage activity was lower in *Sulf1* KO mice without reaching significance over the recording as a whole (Fig. 9b; *p* = 0.11). Analyzed separately by phase, social contact did not differ significantly in either the light or the dark phase (Fig. 9c; *p* = 0.50 and 0.84; *q* = 0.837 and 0.967), whereas activity was reduced during the dark phase but not during the light phase (Fig. 9d; *p* = 0.038 and 0.80). The dark-phase difference did not survive correction within the drug-free family (*q* = 0.244) and is therefore reported as nominal, but its direction and magnitude (2517 versus 1903 arbitrary units, a reduction of 24%) are consistent with the reduced locomotor activity observed in the open field, the three-chamber apparatus, and the home cage before drug injection.

### Home-cage activity and the response to MK-801

**Fig. 10:**
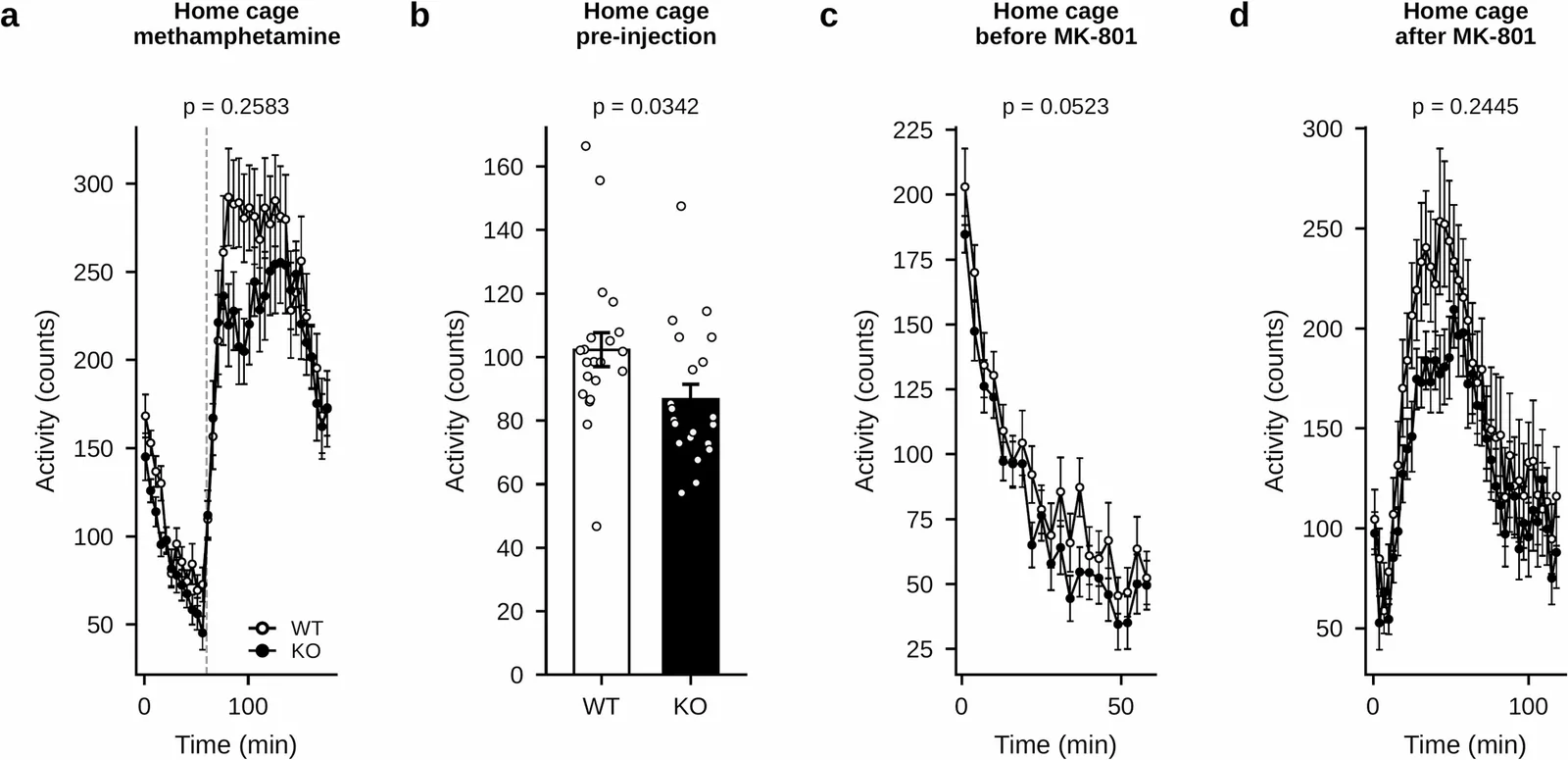
Home-cage activity. (**a**) Activity before and after methamphetamine administration at 60 min (dashed line); the *p* value is for the post-injection period. (**b**) Activity during the 60 min preceding injection. (**c, d**) Activity before and after administration of MK-801 (0.25 mg/kg). Bars show the mean ± SEM and symbols show individual mice

Home-cage activity was recorded before and after administration of methamphetamine or of the NMDA receptor antagonist MK-801 (0.25 mg/kg). Before injection, activity was nominally lower in *Sulf1* KO mice in the methamphetamine session (Fig. 10b; *p* = 0.034, *q* = 0.244) and showed the same tendency in the MK-801 session (Fig. 10c; *p* = 0.052, *q* = 0.248), consistent with the reduced locomotor activity observed in the open field. After injection, neither the response to methamphetamine (Fig. 10a; *p* = 0.26) nor the response to MK-801 (Fig. 10d; *p* = 0.24; *q* = 0.324 and 0.324) differed significantly between genotypes; in the home cage, however, the pre-existing baseline difference and the large drug-induced increase limit the sensitivity of these comparisons.

### Human *SULF1* variation is not supported by large-scale genetic data

Because PPI deficits have been reported in schizophrenia and in bipolar disorder [19, 20], we examined associations between human *SULF1* and these disorders using the methods available at the time of the original study. A transmission disequilibrium test in 128 trios identified a nominal association between rs2704029 and schizophrenia, but this finding was not replicated in either of 2 independent Japanese case-control samples (*p* = 0.43 and 0.97). Analysis of mood disorders yielded a nominal association of rs2704029 with bipolar disorder (*p* = 0.003) but not with depression (*p* = 0.96).

To evaluate these exploratory findings against current data, we examined rs2704029 in publicly available summary statistics from the Psychiatric Genomics Consortium. In the schizophrenia GWAS [13], rs2704029 was not associated with schizophrenia (odds ratio [OR] = 1.007, 95% confidence interval [CI] 0.990–1.024, *p* = 0.45; 74,776 cases and 101,023 controls), and the same was true in the bipolar disorder GWAS [14] (OR = 1.008, 95% CI 0.992–1.023, *p* = 0.33; 67,400 cases and 862,248 controls). Analyses restricted to East Asian samples, matching the ancestry of the present case-control samples, were also non-significant for both disorders (schizophrenia, OR = 1.04, *p* = 0.10; bipolar disorder, OR = 1.05, *p* = 0.19), although these analyses were underpowered. Across the *SULF1* locus (chr8:70,279,016–70,673,147, GRCh37), the minimum *p* value was 1.6 × 10⁻³ for schizophrenia (1,070 variants) and 1.4 × 10⁻⁴ for bipolar disorder (894 variants); neither approached genome-wide significance nor survived correction for the number of variants tested within the region. *SULF1* lies outside all 287 schizophrenia loci [13] and all 298 bipolar disorder loci [14] reported in those studies. The nominal associations described above should therefore be regarded as exploratory and are not supported by current large-scale genetic data.

## Discussion

A comprehensive behavioral analysis of male *Sulf1* KO mice identified 3 phenotypes: reduced spontaneous locomotor activity, a forelimb-selective alteration of gait, and increased acoustic startle responses with reduced PPI. A fourth observation, an attenuated behavioral response to methamphetamine without a significant genotype difference in accumbal dopamine release, suggests a change downstream of dopamine release. Nominal behavioral differences, including increased baseline-subtracted contextual freezing, differences in the number of light/dark transitions, increased revisiting errors in the eight-arm radial maze, and reduced home-cage activity during the dark phase and before methamphetamine injection, are promising findings that warrant confirmation.

### A forelimb-selective gait phenotype

The most robust gait finding was a consistent shift in the temporal pattern of forepaw contact: the proportion of the stance phase preceding maximal paw contact (“brake”) was increased, and the proportion of the stride following maximal contact (“propel”) was reduced. This pattern was present in 2 independent cohorts and at both treadmill speeds tested in the third cohort. Absolute paw angle was increased in the same direction under all 3 conditions, although this measure passed correction for multiple comparisons only at 30 cm/s. Sixteen of the 171 comparisons in the gait family met the false discovery rate threshold; because most of them involve algebraically related measures, or the same underlying variable assessed across cohorts and speeds, they should be read as convergent evidence for altered forepaw contact timing rather than as 16 independent abnormalities. No hindlimb comparison survived correction, and a direct genotype × limb interaction test, performed on the within-animal difference between the 2 limb pairs, was significant for both the timing and the orientation measure in all 3 conditions. The phenotype is therefore selective for the forelimb rather than merely more easily detected there.

Two features of the data constrain the interpretation. First, rotarod performance, grip strength and wire hang latency showed no significant genotype differences, providing no evidence of marked muscular weakness or a gross impairment of balance or motor coordination detectable by these assays; these results do not, however, exclude subtle musculoskeletal, peripheral sensory or sensorimotor abnormalities. Second, the change is not a simple scaling of gait to a lower level of activity, because treadmill speed was imposed rather than chosen by the animal.

The terminology of the DigiGait measures requires cautious interpretation. The “brake” and “propel” phases are defined from the time course of paw contact area — from initial contact to maximal contact, and from maximal contact to paw-off — rather than from direct measurement of ground reaction force [21]. The increased brake fraction is therefore most conservatively interpreted as a delayed progression from initial to maximal forepaw contact, and not as evidence of greater braking force or of reduced propulsive force. Likewise, the increased absolute paw angle indicates a larger deviation of the forepaw from the direction of travel but does not specify the direction of rotation. Such changes could arise from altered limb or paw geometry, from the progression of forelimb loading, from proprioceptive feedback, or from the neural patterning of the step cycle. Because *Sulf1* is deleted in all tissues in this line, forelimb morphology, muscle and joint mechanics, peripheral nerves and proprioceptors, cervical spinal circuits and descending control cannot be distinguished as the site of the change.

The neural level at which *Sulf1* influences gait remains unresolved. Basic forward stepping on a treadmill can be generated by brainstem and spinal locomotor networks, whereas descending cortical and striatal inputs refine the efficiency, stability and adaptability of locomotion. Sulf1 and Sulf2 are jointly required for corticospinal tract formation, and mice lacking both enzymes show abnormal pyramidal decussation, bilateral corticospinal projections, and impaired forelimb reaching [6, 22], whereas no gross corticospinal abnormality has been detected in mice lacking *Sulf1* alone [6]. The severe developmental corticospinal phenotype of the double knockout is therefore an unlikely direct explanation for the present findings. The expression pattern of *Sulf1* does not point to an obvious striatal explanation either. In the adult brain *Sulf*1 is strongly expressed in the shell of the nucleus accumbens, where it is co-expressed with D1 and D2 receptors, and in the tail of the striatum and a periventricular region of the striatum, but it is largely absent from the dorsal striatum [7], the part of the striatum most directly implicated in the control of locomotion. Cervical spinal circuits, proprioceptive afferents and peripheral mechanisms therefore remain equally plausible, and the present data do not distinguish between them.

Segment-level kinematic analysis, electromyographic recording during locomotion, measurement of ground reaction forces, and examination of forelimb anatomy, proprioceptive afferents, and cervical spinal circuits would help to distinguish these possibilities. We also note that gait was measured in 2 of the 3 cohorts and at a single body weight range; whether the phenotype is present in females, and whether it progresses with age, remains to be determined.

Whether the gait phenotype contributes to the reduction in spontaneous locomotion remains unknown. The fixed-speed treadmill results show that the gait difference is not merely a consequence of slower self-selected movement, which is itself informative. However, the brake and propel measures are derived from paw-contact timing rather than from direct measurement of ground reaction force, and stride length and stride frequency showed no evidence of a genotype difference in any condition, so the present data provide no kinematic basis on which to infer a reduction in distance traveled per unit time. The gait and locomotor phenotypes may therefore reflect a common alteration in motor control rather than one directly causing the other.

### Sensorimotor gating

The reduction in PPI is the most consistently replicated of the sensorimotor findings, having been observed in all 5 drug-free or saline-treated sessions across 2 cohorts. The primary drug-free result was nominal after within-family FDR correction (*q* = 0.212). We regard reproduction across independent cohorts as the stronger form of support and have reported both.

Haloperidol was given at 2 doses. Both lie above the threshold of 0.1–0.2 mg/kg at which haloperidol begins to enhance PPI in C57BL/6 mice [23], and the higher dose numerically increased PPI more than the lower one (an increase of 9.4% points over the parallel saline arm at 1 mg/kg, against 3.9% points at 0.3 mg/kg). The genotype difference in PPI was present at both doses. Lower PPI persisted after haloperidol and, at nominal significance, after clozapine; nonsignificant drug × genotype interactions do not establish pharmacological non-responsiveness (Supplementary Table S2). Because PPI in rodents is modulated by dopaminergic, serotonergic, and glutamatergic mechanisms [24], the present data do not identify which system is involved; they do not exclude a contribution of D2 receptor signaling.

### Dissociation between behavior and dopamine release

The attenuated locomotor, vertical and stereotypic responses to methamphetamine occurred without a significant genotype difference in dopamine release in the nucleus accumbens; values were, if anything, larger in *Sulf1* KO mice. The nominally significant drug × genotype interactions, together with the absence of a significant genotype difference in the saline arm for vertical activity and stereotypic counts, support a difference in drug responsiveness beyond the baseline difference in activity. Together with the reported co-expression of Sulf1 with D1 and D2 receptors in the adult brain [7] and the altered reward and aversion learning of these mice [8], the dissociation suggests that Sulf1 acts downstream of dopamine release, at the level of receptor signaling or of the striatal circuits that translate dopamine signals into movement.

### Relation to previous work, and limitations

A previous study reported reduced hippocampal spine density and impaired long-term potentiation in *Sulf1*-deficient mice [9]. We found no genotype difference surviving correction for multiple comparisons in the eight-arm radial maze, the Barnes maze, or contextual fear conditioning. The 2 studies used different knockout lines and different genetic backgrounds, and synaptic changes of this magnitude are not always reflected in the performance of standard spatial tasks; nonetheless, the discrepancy should be kept in mind.

This study has several limitations. First, because we used constitutive *Sulf1* KO mice, we cannot definitively distinguish an ongoing physiological role of *Sulf1* in the adult nervous system from the secondary, persistent consequences of *Sulf1* deficiency during neural development. Given the established functions of *Sulf1/2* in neural development, such as neural differentiation and axon guidance, we cannot fully exclude the possibility that the observed behavioral phenotypes in adulthood are caused by developmental alterations, although no gross neuroanatomical abnormalities have been reported in single *Sulf1* KO mice, including our own analysis [6]. To unequivocally establish an adult-specific physiological requirement for *Sulf1*, future studies utilizing conditional or inducible gene KO models will be necessary. Second, only male mice were examined, so the generality of these findings to females is unknown. Third, the mice were backcrossed for 10 generations, but a congenic interval of 129-derived genome flanking the targeted locus is expected to remain, and a contribution of linked passenger genes cannot be excluded. Fourth, the assignment of measures to families for the purpose of controlling the false discovery rate was made after the experiments were performed and was not preregistered; the sensitivity of the conclusions to this assignment is documented in Supplementary Materials 4. The haloperidol and clozapine experiments included parallel saline arms, permitting exploratory within-session drug × genotype interaction tests (Supplementary Table S2). The 3 sessions used the same cohort in a fixed order, so prior treatment and order effects cannot be excluded. Nonsignificant comparisons do not establish equivalence or exclude biologically meaningful effects. Finally, the human genetic analyses were exploratory, were conducted with the methods available at the time, and are not supported by current large-scale GWAS data.

### Conclusion

Male *Sulf1* KO mice show reduced spontaneous locomotor activity, a reproducible and forelimb-selective alteration of gait, increased acoustic startle responses and reduced PPI, together with an attenuated behavioral response to methamphetamine without a significant genotype difference in accumbal dopamine release. Because we used constitutive *Sulf1* KO mice, we cannot definitively distinguish a physiological role of *Sulf1* in the adult brain from the consequences of *Sulf1* deficiency during development. To unequivocally establish an adult-specific physiological requirement for *Sulf1*, future studies utilizing conditional or inducible KO models will be necessary. Nominal differences in baseline-subtracted contextual freezing, light/dark transitions, radial-maze revisiting errors, and home-cage activity during the dark phase and before methamphetamine injection remain promising candidates for confirmation. The forelimb gait phenotype identifies a specific motor process for future mechanistic study. No significant genotype differences were detected in general health and gross motor measures, elevated plus maze and forced swim measures, Barnes maze measures, social contact measures, or cued fear-conditioning measures.

## Supplementary Information

Below is the link to the electronic supplementary material.

Supplementary Material 1

Supplementary Material 2

Supplementary Material 3

Supplementary Material 4

## Data Availability

No datasets were generated or analysed during the current study.

## Declarations

### Use of AI and AI-assisted technologies

During the preparation of this manuscript, the authors used ChatGPT (OpenAI) and Claude (Anthropic) for language editing, for retrieval and tabulation of publicly available genome-wide association study summary statistics, for statistical computation and cross-checking of analyses performed on author-provided numerical data, and for the preparation of figures from those data. No artificial intelligence tool was used to generate or modify primary experimental data. All outputs were independently verified by the authors, who take full responsibility for the content of this article.

## References

[1] Lamanna WC, Kalus I, Padva M, Baldwin RJ, Merry CL, Dierks T. The heparanome—the enigma of encoding and decoding heparan sulfate sulfation. J Biotechnol. 2007;129(2):290–307. 10.1016/j.jbiotec.2007.01.022 17337080

[2] El Masri R, Seffouh A, Lortat-Jacob H, Vives RR. The in and out of glucosamine 6-O-sulfation: the 6th sense of heparan sulfate. Glycoconj J. 2017;34(3):285–98. 10.1007/s10719-016-9736-5 27812771

[3] Vivès RR, Seffouh A, Lortat-Jacob H. Post-Synthetic Regulation of HS Structure: The Yin and Yang of the Sulfs in Cancer. Front Oncol. 2014;3:331. 10.3389/fonc.2013.00331 PMC3890690 24459635

[4] Ai X, Kitazawa T, Do AT, Kusche-Gullberg M, Labosky PA, Emerson CP Jr. SULF1 and SULF2 regulate heparan sulfate-mediated GDNF signaling for esophageal innervation. Development. 2007;134(18):3327–38. 10.1242/dev.007674 17720696

[5] Holst CR, Bou-Reslan H, Gore BB, Wong K, Grant D, Chalasani S, et al. Secreted sulfatases Sulf1 and Sulf2 have overlapping yet essential roles in mouse neonatal survival. PLoS ONE. 2007;2(6):e575. 10.1371/journal.pone.0000575 PMC1892809 17593974

[6] Okada T, Keino-Masu K, Nagamine S, Kametani F, Ohto T, Hasegawa M, et al. Desulfation of Heparan Sulfate by Sulf1 and Sulf2 Is Required for Corticospinal Tract Formation. Sci Rep. 2017;7(1):13847. 10.1038/s41598-017-14185-3 PMC5653861 29062064

[7] Miya K, Keino-Masu K, Okada T, Kobayashi K, Masu M. Expression of Heparan Sulfate Endosulfatases in the Adult Mouse Brain: Co-expression of Sulf1 and Dopamine D1/D2 Receptors. Front Neuroanat. 2021;15:726718. 10.3389/fnana.2021.726718 PMC8417564 34489650

[8] Miya K, Ohta K, Keino-Masu K, Okada T, Mizuno S, Takahashi S, et al. Essential roles of heparan sulfate endosulfatase Sulf1 in reward and aversion learning through distinct dopamine D1 and D2 receptor pathways in male mice. J Neurochem. 2026;170(1):e70338. 10.1111/jnc.70338 PMC12766148 41486756

[9] Kalus I, Salmen B, Viebahn C, von Figura K, Schmitz D, D’Hooge R, et al. Differential involvement of the extracellular 6-O-endosulfatases Sulf1 and Sulf2 in brain development and neuronal and behavioural plasticity. J Cell Mol Med. 2009;13(11–12):4505–21. 10.1111/j.1582-4934.2008.00558.x PMC4515066 20394677

[10] Takao K, Tanda K, Nakamura K, Kasahara J, Nakao K, Katsuki M, et al. Comprehensive behavioral analysis of calcium/calmodulin-dependent protein kinase IV knockout mice. PLoS ONE. 2010;5(3):e9460. 10.1371/journal.pone.0009460 PMC2830479 20209163

[11] Takao K, Yamasaki N, Miyakawa T. Impact of brain-behavior phenotyping of genetically-engineered mice on research of neuropsychiatric disorders. Neurosci Res. 2007;58(2):124–32. 10.1016/j.neures.2007.02.009 17524507

[12] Benjamini Y, Hochberg Y. Controlling the false discovery rate: a practical and powerful approach to multiple testing. J R Stat Soc Ser B. 1995;57(1):289–300.

[13] Trubetskoy V, Pardiñas AF, Qi T, Panagiotaropoulou G, Awasthi S, Bigdeli TB, et al. Mapping genomic loci implicates genes and synaptic biology in schizophrenia. Nature. 2022;604(7906):502–8. 10.1038/s41586-022-04434-5 PMC9392466 35396580

[14] O’Connell KS, Koromina M, van der Veen T, Boltz T, David FS, Yang JMK, et al. Genomics yields biological and phenotypic insights into bipolar disorder. Nature. 2025;639(8056):968–75. 10.1038/s41586-024-08468-9 PMC12163093 39843750

[15] Nagamine S, Tamba M, Ishimine H, Araki K, Shiomi K, Okada T, et al. Organ-specific sulfation patterns of heparan sulfate generated by extracellular sulfatases Sulf1 and Sulf2 in mice. J Biol Chem. 2012;287(12):9579–90. 10.1074/jbc.M111.290262 PMC3308746 22298771

[16] Sato DX, Chatzigiannis MM, Shoji H, Sala G, Hattori S, Takao K, et al. Large-scale dimensional behavioral profiling dissociates fear memory from locomotor confounds in mice: the necessity of baseline-normalized metrics. bioRxiv. 2026. 10.64898/2026.07.12.738100.

[17] Hagino Y, Kasai S, Han W, Yamamoto H, Nabeshima T, Mishina M, et al. Essential role of NMDA receptor channel epsilon4 subunit (GluN2D) in the effects of phencyclidine, but not methamphetamine. PLoS ONE. 2010;5(10):e13722. 10.1371/journal.pone.0013722 PMC2965660 21060893

[18] Miyakawa T, Leiter LM, Gerber DJ, Gainetdinov RR, Sotnikova TD, Zeng H, et al. Conditional calcineurin knockout mice exhibit multiple abnormal behaviors related to schizophrenia. Proc Natl Acad Sci U S A. 2003;100(15):8987–92. 10.1073/pnas.1432926100 PMC166425 12851457

[19] Braff D, Stone C, Callaway E, Geyer M, Glick I, Bali L. Prestimulus effects on human startle reflex in normals and schizophrenics.Psychophysiology. 1978;15(4):339–43. 10.1111/j.1469-8986.1978.tb01390.x 693742

[20] Perry W, Minassian A, Feifel D, Braff DL. Sensorimotorgating deficits in bipolar disorder patients with acute psychotic mania. BiolPsychiatry. 2001;50(6):418–24. 10.1016/s0006-3223(01)01184-2 11566158

[21] Amende I, Kale A, McCue S, Glazier S, Morgan JP, Hampton TG. Gait dynamics in mouse models of Parkinson’s disease and Huntington’s disease. J Neuroeng Rehabil. 2005;2:20. 10.1186/1743-0003-2-20 PMC1201165 16042805

[22] Aizawa S, Okada T, Keino-Masu K, Doan TH, Koganezawa T, Akiyama M, et al. Abnormal pyramidal decussation and bilateral projection of the corticospinal tract axons in mice lacking the heparan sulfate endosulfatases, Sulf1 and Sulf2. Front Mol Neurosci. 2020;12:333. 10.3389/fnmol.2019.00333 PMC6985096 32038163

[23] McCaughran J Jr, Mahjubi E, Decena E, Hitzemann R.Genetics, haloperidol-induced catalepsy and haloperidol-induced changes in acoustic startle and prepulse inhibition. Psychopharmacology. 1997;134(2):131–9. 10.1007/s002130050434 9399376

[24] Zhang J, Forkstam C, Engel JA, Svensson L. Role ofdopamine in prepulse inhibition of acoustic startle. Psychopharmacology (Berl).2000;149(2):181–8. 10.1007/s002130000369 10805614

