# Supplementary material for: Comprehensive behavioral phenotyping of male Sulf1-deficient mice reveals reduced locomotor activity, altered forelimb gait, and reduced prepulse inhibition
Source: Mol Brain. 2026 Oct 17;19:73. doi: 10.1186/s13041-026-01353-8 (PMC13681803; doi:10.1186/s13041-026-01353-8)
Supplement: Supplementary file 1 [file 13041_2026_1353_MOESM1_ESM.docx]

**Supplementary Materials 1**

**Methods**

**Animals**

All animal experiments were approved by and performed according to the guidelines of the animal care and use committees of the University of Tsukuba, Kyoto University, and Tokyo Institute of Psychiatry (now integrated into the Tokyo Metropolitan Institute of Medical Science). Mice were maintained under standard conditions with a 12-h light/dark cycle and free access to water and food. The mouse strain used was *Sulf1* KO mice (MGI 5318489) [1] that had been backcrossed to C57BL/6N for 10 successive generations. Genotypes were determined by use of genomic PCR with tail biopsy.

**Behavioral tests**

Male mice were subjected to behavioral experiments in the order listed below, as described previously [2]. Behavioral experiments began at 10 weeks of age. The experiments were conducted in 3 different batches. The numbers of total mice used in this study were 73 for the control WT and 73 for the *Sulf1* KO mice.

**Light/dark transition test**

The light/dark transition test was conducted by use of a cage (21×42×25 cm) divided into 2 chambers of equal size (O'Hara & Co., Tokyo, Japan). One chamber was brightly illuminated (390 lux) and the other darkly (2 lux). A mouse was placed into the dark chamber and allowed to move freely for 10 min. Distance traveled, stay time in the light chamber, transitions between 2 chambers, and latency to enter the light chamber were analyzed.

**Open field test**

The open field test was conducted by use of an apparatus (40×40×30 cm; Accuscan Instruments, Columbus, OH, USA). A mouse was placed in the square arena and allowed to explore freely for 30­–120 min. Vertical activity was detected by photobeam interruption. Total distance traveled, vertical activity, time spent in the center area, and stereotypic counts were analyzed. In some experiments, methamphetamine (3 mg/kg) or saline was intraperitoneally administered to mice 60 min after they had entered the arena, and they were observed for a further 120 min after drug administration. Methamphetamine- and saline-treated groups were run in parallel in the same session.

**Elevated plus maze test**

The elevated plus maze test was conducted by use of an apparatus consisting of 2 open arms (25×5 cm) and 2 closed arms (25×5 cm) with 15-cm high transparent walls. A mouse was placed in the central square and allowed to move freely for 10 min. The number of total entries, entry into open arms, total distance traveled, and time spent in the open arms were analyzed.

**Hot plate test**

A mouse was placed on a 55.0 ℃ hot plate (Columbus Instruments, Columbus, OH, USA) and latency to foot shaking or paw licking was analyzed.

**Social interaction test in a novel environment**

Two mice of the same genotype that had been housed in different cages were placed in a chamber (40×40×30 cm) and allowed to move freely for 10 min. Total duration of contact, number of contacts, total duration of active contact, mean contact duration, and total distance traveled were analyzed.

**Rotarod test**

The rotarod test was performed by use of an accelerating rotarod apparatus (Ugo Basile, Lombardy, Italy). A mouse was placed on a rotating drum (3 cm diameter) and latency to fall from the drum was measured. The rotational speed was increased from 4 to 40 rpm over a 5-min period.

**Prepulse inhibition test**

The prepulse inhibition test was conducted by use of a measurement system (O'Hara & Co.). A mouse that had been put into a Plexiglas cylinder was placed in the apparatus and startle responses to loud sound were analyzed. The duration of startle stimuli (110 or 120 dB) was 40 ms, and the level of background noise was kept at 70 dB. The prepulse stimulus of 74 or 78 dB was given 100 ms before the startle stimuli. Six blocks of 6 trial types (2 types of startle stimulus only, and 4 types of combinations of prepulse and startle stimuli: 74–110 dB, 78–110 dB, 74–120 dB, and 78–120 dB) were presented in random order. The average intertrial interval was 15 s. In some experiments, haloperidol (0.3 or 1 mg/kg) or clozapine (3 mg/kg) was administered intraperitoneally before the start of the experiment. In each drug session, a saline-treated group was run in parallel. The drug sessions were conducted in the order haloperidol 0.3 mg/kg, clozapine 3 mg/kg, haloperidol 1 mg/kg, using the same cohort of animals. Each session included WT–saline, KO–saline, WT–drug, and KO–drug groups (*n* = 15 per group), with treatment assignments changing across sessions.

**Porsolt forced swim test**

A mouse was placed in a cylinder (20-cm height×10-cm diameter) filled with water (23 ℃), and its behavior was recorded for 10 min. Immobility time and total distance traveled were analyzed.

**Radial maze test**

The eight-arm radial maze test was conducted by use of an apparatus (O'Hara & Co.) consisting of an octagonal central platform and 8 arms (9×40 cm). Food wells with pellet sensors were placed at the distal end of each arm. After pretraining trials, maze acquisition trials were performed: after the 8 arms were baited with food pellets, a mouse was placed on the central platform and allowed to obtain the 8 pellets within 25 min. The number of different arm choices during the first 8 entries and the total number of arms revisited were recorded. In trials 30–35, when 4 pellets were obtained, a delay (30, 120, or 300 s) was applied. One WT mouse died during the acquisition phase, after completing 16 of the 35 trials, and was excluded from the analysis of this test; the analysed set was therefore 21 WT and 22 KO mice. The same animal had completed the Barnes maze test (22 WT and 22 KO mice) and was absent from the subsequent gait analysis.

**Barnes maze test**

The Barnes maze test was conducted by use of a white circular platform (1.0 m in diameter) with 12 holes (O'Hara & Co.). A black escape box was located under one of the 12 holes. The location of the target relative to the visual cues in the room was kept unchanged for a given mouse. Three trials per day were conducted for 6 days. On days 7 and 14, a probe test was conducted without the escape box to evaluate memory retention.

**Contextual and cued fear conditioning test**

On day 1, a mouse was placed in a test chamber (26×34×39 cm) and allowed to explore freely for 2 min. After a white noise (60 dB) was presented for 30 s as the conditioned stimulus (CS), a mild foot shock (0.5 mA, 2 s) was given as the unconditioned stimulus (US). Two more combinations of CS-US pairing were presented twice at 2-min intervals. Context testing was performed by use of the same chamber 24 h and 8 d after conditioning. Cued testing was performed by use of a different chamber (illuminated at 100 lux) after habituation to this chamber. To examine long-term fear memory, cued testing was performed on days 2, 9, 10, and 13. In each test, percentages of freezing, obtained from image analysis, were measured.

**Crawley's sociability and preference for social novelty test**

The test for sociability and preference for social novelty was conducted as previously described [3,4]. The apparatus comprised a rectangular, three-chambered box and a lid containing an infrared video camera (O'Hara & Co.). Each chamber was 20 × 40 × 22 cm and the dividing walls were made from clear Plexiglas, with small square openings (5 × 3 cm) allowing access into each chamber. An unfamiliar C57BL/6J male (stranger 1) that had no prior contact with the subject mouse was placed in one of the side chambers. The placement of stranger 1 in the left or right side chamber was systematically alternated between trials. The stranger mouse was enclosed in a small, circular wire cage that allowed nose contact between the bars but prevented fighting. The cage was 11 cm high, with a bottom diameter of 9 cm and bars spaced 0.5 cm apart. The subject mouse was first placed in the middle chamber and allowed to explore the entire social test box for 10 min. The amount of time spent within a 5-cm distance of the wire cage and in each chamber was recorded. At the end of the first 10 min, each mouse was tested in a second 10-min session to quantify social preference for a new stranger. A second, unfamiliar mouse was placed in the chamber that had been empty during the first session, enclosed in an identical small wire cage. The test mouse thus had a choice between the already-investigated unfamiliar mouse (stranger 1) and the novel unfamiliar mouse (stranger 2), and the time spent within a 5-cm distance of each wire cage and in each chamber during the second session was recorded. The stranger mice were 8- to 12-week-old C57BL/6J males that were not littermates. Analysis was performed automatically using Image CSI software (O'Hara & Co., Tokyo, Japan).

**Gait analysis**

Gait was analyzed during walk/trot locomotion by ventral plane videography using the DigiGait Imaging System (Mouse Specifics Inc., Boston, MA, USA). The system allows mice to walk on a motorized transparent treadmill belt while digital video images of the underside of the animal are collected at 150 frames per second, and the software automatically identifies the stance and swing components of each stride. The apparatus has 4 chambers, and 4 mice were run in parallel in each trial. The second cohort was tested in April 2006 at a belt speed of 24.7 cm/s. The third cohort was tested in December 2006 at 2 belt speeds, 20 and 30 cm/s, which were recorded on 2 separate days. Measures were obtained separately for the forelimbs and the hindlimbs. Within a stride, the stance phase was divided into a braking phase, from the first contact of the paw with the belt until the paw was maximally in contact, and a propulsion phase, from that point until the paw left the belt; the swing phase is the remainder of the stride. Measures are reported both as absolute durations and as percentages of the stance or of the stride. Paw angle is the angle between the axis of the paw and the direction of travel, and absolute paw angle is its magnitude irrespective of sign. The braking and propulsion phases are therefore defined from the time course of the paw contact area recorded by the video system; the DigiGait system does not measure ground reaction force, and these phases should not be interpreted as measurements of braking or propulsive force. All measures returned by the software were analysed. Twenty-eight measures are returned for the forelimb and twenty-nine for the hindlimb, Hind Limb Shared Stance Time being defined for the hindlimb only, giving (28 + 29) × 3 conditions = 171 comparisons across cohorts, belt speeds and limb pairs. Values for the left and right paws were averaged within each limb pair by the software, and the individual mouse was the unit of analysis. In the second cohort, 27 WT and 28 KO mice were analysed; in the third cohort, 19 WT and 22 KO mice at 20 cm/s and 21 WT and 22 KO mice at 30 cm/s. One WT mouse of the third cohort died before the gait recordings and was therefore not tested at either belt speed; two further WT mice of that cohort did not yield a complete set of measures at 20 cm/s and were omitted from that condition only. The number of steps contributing to each animal’s values, as returned by the software, had a median of 10 (range 6–14). Because the 2 limb pairs were recorded from the same animals in the same run, the forelimb and hindlimb values are paired within each mouse, which permits a direct test of whether the genotype effect differs between limbs (see Statistical analysis).

**Social interaction test in home cage**

Two mice of the same genotype that had been housed in different cages were placed in a chamber (29×18×12 cm) and their behavior was recorded continuously with an infrared video camera for 1 week [5]. Images were captured at 2-min intervals and binned into 1-h bins, each labeled by its start time; the recording runs started at different wall-clock times and were aligned on clock time. Social contact was indexed by the mean number of particles detected per frame, 1 particle indicating that the 2 mice were resolved as a single object because they were touching and 2 particles indicating that they were apart. Locomotor activity was measured in parallel as the number of pixels that changed between successive frames and is expressed in arbitrary units. Eight cage pairs per genotype were recorded. The cage pair, not the individual mouse and not the individual time bin, was the unit of analysis, because bins within a cage are not independent. The first 24 h were discarded as a habituation period, and the remaining recording was analyzed; the light phase was defined as the bins from 07:00 to 18:00 and the dark phase as those from 19:00 to 06:00, corresponding to lights on at 07:00 and off at 19:00. One recording run suffered a 13-h outage on day 5 that affected 2 WT and 2 KO cages equally; for the reported genotype comparisons, time bins with a missing value in any cage were omitted for all cages, retaining all 8 cage pairs per genotype.

**Home-cage activity with drug challenge**

Locomotor activity in the home cage was recorded before and after administration of methamphetamine or of the NMDA receptor antagonist MK-801, using the home-cage monitoring system described above and following the design of Miyakawa et al. [5]. The cages contained no bedding, water bottle or food during the recording. Each mouse was placed in a cage and activity was recorded for 65 min as a habituation period, with images binned at 1-min intervals. The mouse was then removed briefly, injected intraperitoneally, returned to the same cage, and activity was recorded for a further 125 min. MK-801 was dissolved in saline and given at 0.25 mg/kg (0.3 ml of a 0.025 mg/ml solution for a 30-g mouse); methamphetamine was given at 3 mg/kg. For analysis, the last 60 min of the habituation period and the first 60 min after injection were used, and the ratio of post-injection to pre-injection activity was computed for each mouse, as in Miyakawa et al. [5].

**In vivo microdialysis**

In vivo microdialysis was conducted as reported previously [6]. Mice were implanted with microdialysis probes under anesthesia with intraperitoneal injection of 50 mg/kg sodium pentobarbital. The stereotaxic coordinates were as follows: anteroposterior +1.2 mm, mediolateral +0.5 mm, and dorsoventral -5.0 mm from the bregma. The probe tip (outer diameter of 0.22 mm, length of 1 mm) was constructed with a regenerated cellulose membrane (Eicom, Kyoto, Japan). All dialysis probe placements were verified histologically at the completion of the experiment. Twenty-four hours after probe implantation, dialysis was performed by use of freely-moving mice. Ringer's solution (145 mM NaCl, 3 mM KCl, 1.26 mM CaCl_2_, and 1 mM MgCl_2_, pH 6.5) was perfused at a constant flow rate of 1 µl/min. Perfusates were injected directly into the high-performance liquid chromatography system every 10 min by use of an autoinjector (EAS-10, Eicom). Dialysate dopamine and serotonin were separated by reverse-phase ODS column chromatography (PP-ODS, Eicom) and detected with a graphite electrode (HTEC-500, Eicom). The mobile phase consisted of 0.1 M phosphate buffer (pH5.5) containing 500 mg/l sodium decanesulfonate, 50 mg/l EDTA, and 1% methanol. After the baseline samples were collected for 180 min, methamphetamine (1 mg/kg) or saline was subcutaneously administered and measurements were continued for further 180 min.

**Human genetic association study**

A transmission disequilibrium test was performed for 128 trios consisting of probands with schizophrenia and their parents as described previously [7]. Association between the *SULF1* gene and schizophrenia was assessed as described previously [8]. The screening groups comprised 570 unrelated Japanese patients diagnosed with schizophrenia and 566 mentally healthy Japanese controls. The replication groups comprised 1334 unrelated Japanese patients with schizophrenia and 1339 mentally healthy Japanese controls. Written informed consent was obtained from all the participants. The study was approved by the ethics committees of the University of Tsukuba, Niigata University, Fujita Health University, Nagoya University, Okayama University, and Seiwa Hospital. Association between the *SULF1* gene and bipolar disorders was assessed as described previously [9]. The participants were 149 unrelated Japanese patients with bipolar disorders, 152 unrelated Japanese patients with depressive disorders, and 1557 control participants. Written informed consent was obtained from all the participants. The study was approved by the ethics committees of the University of Tsukuba, RIKEN, Tokyo Medical and Dental University, and the National Institute of Mental Health (NIMH). Allelic and genotypic associations were evaluated by use of the Fisher exact test and the Cochran-Armitage trend test, respectively.

**Statistical analysis**

Statistical analysis of behavioral tests was conducted by use of StatView (SAS Institute, Cary, NC, USA) and SPSS Statistics ver. 30 (IBM, Somers, NY, USA). The reanalysis performed for the present article, including the Benjamini–Hochberg adjustment, the genotype × limb interaction and the figures, used Python 3.12 with NumPy, SciPy, statsmodels, pandas and Matplotlib. Data were analyzed with a two-tailed *t*-test, two-way ANOVA, two-way repeated measures ANOVA, or two-way mixed model ANOVA. Values in graphs are expressed as means ± SEM. Because a large number of behavioral measures was examined, the false discovery rate was controlled at 5% within each group of tests ("test family") by the Benjamini–Hochberg procedure [10]. The 57 drug-free genotype comparisons listed in Supplementary Table S1a were treated as one family of tests, and those obtained from the hypothesis-driven follow-up experiments (methamphetamine challenge, haloperidol and clozapine challenges with their parallel saline arms, and in vivo microdialysis) as a second family (30 measures). The two Day-1 baseline comparisons in fear conditioning and the 4 delayed-trial comparisons in the eight-arm radial maze were not included in Family 1 and are reported with unadjusted *p* values. The measures obtained from the home-cage social interaction test, including those computed separately for the light and the dark phase, and the pre-injection measures of the home-cage drug challenges, belong to the first family, whereas the post-injection measures of those challenges belong to the second. The phase-split home-cage social interaction measures are a post hoc decomposition of measures already in the first family, and both are counted, which is the conservative choice. Gait analysis, which was performed on a separate apparatus in different cohorts and yields a large set of internally redundant measures, was treated as a third family (171 comparisons; see Supplementary Materials 2 and Supplementary Data 1). Genotype effects on gait measures were tested by two-tailed Student *t*-tests. Whether the genotype effect differed between the forelimb and the hindlimb was tested on the within-animal difference score *D* = forelimb value − hindlimb value, compared between genotypes by a two-tailed *t*-test; this is equivalent to the genotype × limb interaction of a mixed-model analysis of variance with limb as the within-subject factor. The interaction was tested for %BrakeStance and for absolute paw angle in each cohort and at each belt speed, and was repeated after logit and log transformation respectively as a sensitivity analysis. For repeated-measures data, a single *p* value for the genotype main effect was used and counted as one test. For the home-cage social interaction test, the reported value is the genotype main effect of a two-way repeated measures ANOVA with genotype as the between-subject factor and time bin as the within-subject factor, which tests genotype against the between-cage error stratum; a Welch *t*-test on per-cage phase means gave closely similar values. A measure with an adjusted *p* value (*q*) below 0.05 within its family is described as significant after within-family FDR correction, and a measure significant only before adjustment is described as nominal. All comparisons included in Families 1 and 2, with raw and adjusted *p* values, are listed in Supplementary Table S1 (Supplementary Materials 2). The complete list of the gait comparisons in Family 3, with group means, effect estimates, 95% confidence intervals, and raw and adjusted *p* values, is provided as Supplementary Data 1, and the per-animal numerical source data for all figures and tests are provided as Supplementary Data 2. Exploratory drug × genotype interactions were tested separately for each antipsychotic session by two-way ANOVA of per-animal means (startle amplitude averaged across the 110- and 120-dB stimuli; PPI averaged across the 74- and 78-dB prepulses separately for each startle intensity). The 9 interaction *p* values were adjusted together by the Benjamini–Hochberg procedure as a separate exploratory family. Effect estimates and pointwise 95% confidence intervals are reported in Supplementary Materials 2, Section 8a and Table S2.

**References**

1. Nagamine S, Tamba M, Ishimine H, Araki K, Shiomi K, Okada T, et al. Organ-specific sulfation patterns of heparan sulfate generated by extracellular sulfatases Sulf1 and Sulf2 in mice. J Biol Chem. 2012;287(12):9579–90.

2. Takao K, Tanda K, Nakamura K, Kasahara J, Nakao K, Katsuki M, et al. Comprehensive behavioral analysis of calcium/calmodulin-dependent protein kinase IV knockout mice. PLoS One. 2010;5(3):e9460.

3. Moy SS, Nadler JJ, Perez A, Barbaro RP, Johns JM, Magnuson TR, et al. Sociability and preference for social novelty in five inbred strains: an approach to assess autistic-like behavior in mice. Genes Brain Behav. 2004;3(5):287–302.

4. Tanda K, Nishi A, Matsuo N, Nakanishi K, Yamasaki N, Sugimoto T, et al. Abnormal social behavior, hyperactivity, impaired remote spatial memory, and increased D1-mediated dopaminergic signaling in neuronal nitric oxide synthase knockout mice. Mol Brain. 2009;2:19.

5. Miyakawa T, Leiter LM, Gerber DJ, Gainetdinov RR, Sotnikova TD, Zeng H, et al. Conditional calcineurin knockout mice exhibit multiple abnormal behaviors related to schizophrenia. Proc Natl Acad Sci USA. 2003;100(15):8987–92.

6. Hagino Y, Kasai S, Han W, Yamamoto H, Nabeshima T, Mishina M, et al. Essential role of NMDA receptor channel epsilon4 subunit (GluN2D) in the effects of phencyclidine, but not methamphetamine. PLoS One. 2010;5(10):e13722.

7. Ohtsuki T, Horiuchi Y, Koga M, Ishiguro H, Inada T, Iwata N, et al. Association of polymorphisms in the haplotype block spanning the alternatively spliced exons of the NTNG1 gene at 1p13.3 with schizophrenia in Japanese populations. Neurosci Lett. 2008;435(3):194–7.

8. Horiuchi Y, Iida S, Koga M, Ishiguro H, Iijima Y, Inada T, et al. Association of SNPs linked to increased expression of SLC1A1 with schizophrenia. Am J Med Genet B Neuropsychiatr Genet. 2012;159B(1):30–7.

9. Horiuchi Y, Nakayama J, Ishiguro H, Ohtsuki T, Detera-Wadleigh SD, Toyota T, et al. Possible association between a haplotype of the GABA-A receptor alpha 1 subunit gene (GABRA1) and mood disorders. Biol Psychiatry. 2004;55(1):40–5.

10. Benjamini Y, Hochberg Y. Controlling the false discovery rate: a practical and powerful approach to multiple testing. J R Stat Soc Series B. 1995;57(1):289–300.
