# Supplementary material for: Comprehensive behavioral phenotyping of male Sulf1-deficient mice reveals reduced locomotor activity, altered forelimb gait, and reduced prepulse inhibition
Source: Mol Brain. 2026 Oct 17;19:73. doi: 10.1186/s13041-026-01353-8 (PMC13681803; doi:10.1186/s13041-026-01353-8)
Supplement: Supplementary file 2 [file 13041_2026_1353_MOESM2_ESM.docx]

**Supplementary Materials 2**

**Correction for multiple comparisons across the behavioral test battery**

*Keino-Masu et al., Comprehensive behavioral phenotyping of male Sulf1-deficient mice*

# **1 Rationale**

A comprehensive behavioral test battery generates a large number of dependent measures from the same cohorts of animals. Reporting each measure against an uncorrected threshold of 0.05 inflates the expected number of false positives in proportion to the number of measures examined. We therefore defined a correction procedure and a criterion for significance after within-family false discovery rate (FDR) correction, and we report every comparison included in these families, irrespective of significance.

# **2 Definition of test families**

The comparisons included in the multiplicity correction were assigned to one of 3 families.

*Family 1 — drug-free test battery.* The 57 drug-free genotype comparisons listed in Supplementary Table S1a: general health examination, rotarod, hot plate, open field, light/dark transition, elevated plus maze, Porsolt forced swim, acoustic startle response and prepulse inhibition, eight-arm radial maze, Barnes maze, social interaction in a novel environment, Crawley's three-chamber test, contextual and cued fear conditioning (conventional and baseline-normalized indices), home-cage social interaction (whole recording and the light and dark phases separately), and home-cage activity before drug injection in the methamphetamine and MK-801 challenges. The phase-split home-cage measures are a post hoc decomposition of measures already in the family; both are counted, which is the conservative choice. This family constitutes the untargeted screen in which discovery occurred.

The two Day-1 baseline comparisons in fear conditioning and the 4 delayed-trial comparisons in the eight-arm radial maze were not included in Family 1 and are reported with unadjusted *p* values.

*Family 2 — hypothesis-driven follow-up experiments.* Measures obtained from experiments performed to follow up findings of the screen: the methamphetamine challenge in the open field, the haloperidol and clozapine challenges in the startle and prepulse inhibition test, the saline arms run in parallel with each drug session, the post-injection home-cage measures of the methamphetamine and MK-801 challenges, and in vivo microdialysis. The 3 antipsychotic sessions used the same cohort, with parallel saline and drug groups in each session. The statistical family assignment was made retrospectively.

*Family 3 — gait analysis.* Gait was measured on a separate apparatus, in different cohorts, and yields a large set of internally redundant measures (for example, the percentage of stance spent braking and the percentage spent propelling are close to complementary by definition). Correcting these jointly with the screen would let the accounting of the gait measures determine the outcome for unrelated tests, so they were treated as a separate family.

# **3 Procedure**

Within each family, raw *p* values for the genotype effect were adjusted by the Benjamini–Hochberg procedure controlling the false discovery rate at 5% (Benjamini and Hochberg, J R Stat Soc B 1995;57:289–300). Repeated-measures data (time bins within a session, trials within a task) contributed a single *p* value for the genotype main effect and were counted as one test, not one test per bin.

A measure is described as *significant after within-family FDR correction* when its Benjamini–Hochberg adjusted *p* value (*q*) is below 0.05. A measure with an unadjusted *p* value below 0.05 but q ≥ 0.05 is described as nominal. Each *q* value refers to the specified family of *tests.*

The Benjamini–Hochberg procedure controls the FDR under independence or suitable positive dependence. Correlation among behavioral measures does not by itself establish that the formal positive-dependence condition is satisfied.

# **4 Supplementary Table S1a. Family 1 — drug-free test battery (m = 57)**

| **Test / domain** | **Measure** | **Figure** | **n (WT/KO)** | **Raw p** | **q** | **q < 0.05** |
| --- | --- | --- | --- | --- | --- | --- |
| Barnes maze | Distance traveled | 6h | 22/22 | 0.0725 | 0.315 |  |
|  | Primary errors | 6f, g | 22/22 | 0.6103 | 0.905 |  |
|  | Primary latency | 6f, g | 22/22 | 0.5359 | 0.849 |  |
|  | Probe test, day 14 (time around target) | 6i, j | 22/22 | 0.6241 | 0.905 |  |
|  | Probe test, day 7 (time around target) | 6i, j | 22/22 | 0.6900 | 0.905 |  |
| Eight-arm radial maze | Different arm choices in first 8 entries | 6a, b | 21/22 | 0.1543 | 0.463 |  |
|  | Distance traveled | 6c | 21/22 | 0.8778 | 0.970 |  |
|  | Latency to take 8 pellets | 6d | 21/22 | 0.1525 | 0.463 |  |
|  | Number of omission errors | 6e | 21/22 | 0.8485 | 0.967 |  |
|  | Number of revisiting errors | 6a, b | 21/22 | 0.0485 | 0.248 |  |
| Elevated plus maze | Distance traveled | 1k–n | 21/21 | 0.5143 | 0.837 |  |
|  | Entries into open arms (%) | 1k–n | 21/21 | 0.0774 | 0.315 |  |
|  | Number of total arm entries | 1k–n | 21/21 | 1.0000 | 1.000 |  |
|  | Time in open arms (%) | 1k–n | 21/21 | 0.3178 | 0.675 |  |
| Fear conditioning | Activity suppression ratio, context | 8g | 21/21 | 0.2128 | 0.568 |  |
|  | Activity suppression ratio, cued | 8h | 21/21 | 0.3381 | 0.678 |  |
|  | Freezing subtraction, context | 8e | 21/21 | 0.0041 | 0.058 |  |
|  | Freezing subtraction, cued | 8f | 21/21 | 0.7787 | 0.944 |  |
|  | Freezing, conditioning (day 1) | 8a, b | 21/21 | 0.0480 | 0.248 |  |
|  | Freezing, context test | 8a, b | 21/21 | 0.0329 | 0.244 |  |
|  | Freezing, cued test | 8c | 21/21 | 0.2375 | 0.589 |  |
| General health | Body temperature | 1a–d | 16/16 | 0.9401 | 0.970 |  |
|  | Body weight | 1a–d | 16/16 | 0.7302 | 0.905 |  |
|  | Grip strength | 1a–d | 16/16 | 0.2840 | 0.648 |  |
|  | Wire hang latency | 1a–d | 16/16 | 0.6388 | 0.905 |  |
| Home-cage activity | Activity before injection (MK-801 session) | 10c | 20/20 | 0.0523 | 0.248 |  |
|  | Activity before injection (methamphetamine session) | 10b | 21/21 | 0.0342 | 0.244 |  |
| Home-cage social interaction | Activity level, whole recording | 9b | 8/8 | 0.1103 | 0.393 |  |
|  | Activity, dark phase | 9d | 8/8 | 0.0385 | 0.244 |  |
|  | Activity, light phase | 9d | 8/8 | 0.7964 | 0.946 |  |
|  | Contact, dark phase | 9c | 8/8 | 0.8411 | 0.967 |  |
|  | Contact, light phase | 9c | 8/8 | 0.4959 | 0.837 |  |
|  | Mean number of particles (contact), whole recording | 9a | 8/8 | 0.6740 | 0.905 |  |
| Hot plate | Latency to response | 1f | 21/21 | 0.3452 | 0.678 |  |
| Light/dark transition | Distance traveled | 1g–j | 21/21 | 0.1261 | 0.423 |  |
|  | Latency to enter light | 1g–j | 21/21 | 0.1980 | 0.564 |  |
|  | Number of transitions | 1g–j | 21/21 | 0.0275 | 0.244 |  |
|  | Stay time in light chamber | 1g–j | 21/21 | 0.2507 | 0.595 |  |
| Open field | Center time | 2a–h | 30/29 | 0.6994 | 0.905 |  |
|  | Stereotypic counts | 2a–h | 30/29 | 0.4068 | 0.748 |  |
|  | Total distance traveled | 2a–h | 30/29 | 0.0023 | 0.044 | **YES** |
|  | Vertical activity | 2a–h | 30/29 | 0.8849 | 0.970 |  |
| Porsolt forced swim | Distance traveled | 1p | 21/21 | 0.5012 | 0.837 |  |
|  | Immobility | 1o | 21/21 | 0.9466 | 0.970 |  |
| Rotarod | Latency to fall | 1e | 21/21 | 0.7213 | 0.905 |  |
| Social interaction (novel environment) | Distance traveled | 7a–d | 10/10 | 0.3196 | 0.675 |  |
|  | Mean contact duration | 7a–d | 10/10 | 0.9236 | 0.970 |  |
|  | Number of contacts | 7a–d | 10/10 | 0.7115 | 0.905 |  |
|  | Total duration of active contact | 7a–d | 10/10 | 0.4829 | 0.837 |  |
|  | Total duration of contact | 7a–d | 10/10 | 0.7124 | 0.905 |  |
| Startle / PPI | PPI, 110-dB startle | 5b, c | 16/16 | 0.2193 | 0.568 |  |
|  | PPI, 120-dB startle | 5b, c | 16/16 | 0.0186 | 0.212 |  |
|  | Startle amplitude | 5a | 16/16 | 0.0004 | 0.023 | **YES** |
| Three-chamber test | Distance traveled | 7g | 21/21 | 0.0013 | 0.037 | **YES** |
|  | Stay time, center area | — | 21/21 | 0.3633 | 0.690 |  |
|  | Stay time, left cage | — | 21/21 | 0.9531 | 0.970 |  |
|  | Stay time, right cage | — | 21/21 | 0.0975 | 0.370 |  |

Figure, panel of the main text in which the measure is plotted. Raw *p* values are for the genotype effect; for repeated-measures data the value is the genotype main effect, computed as a *t*-test on per-animal means across bins or trials, which is algebraically identical to the between-subject main effect of a two-way repeated-measures ANOVA in a balanced design. *q*, Benjamini–Hochberg adjusted *p* value within family. The *q* < 0.05 column indicates significance after within-family FDR correction.

Where a measure was obtained in more than one cohort, the entry corresponds to the cohort plotted in the figures; results from the remaining cohorts are reported in Section 7 as replication and are not counted as separate tests. For the Porsolt forced swim test, which was conducted on two consecutive days, the entry is the per-animal mean of the two test days (*n* = 21 per genotype), so that each mouse contributes one value; the two days are plotted separately in Fig. 1o and 1p.

For the three-chamber test, each value is the per-animal mean across the 2 sessions. Left and right refer to physical cage positions, not cage contents. The 3 stay-time comparisons are not plotted in Figure 7 and are therefore marked with a dash in the Figure column.

The two Day-1 baseline comparisons in fear conditioning (freezing, *p* = 0.0087; distance traveled, *p* = 0.53) and the 4 delayed-trial comparisons in the eight-arm radial maze (different arm choices, *p* = 0.19; revisiting errors, *p* = 0.52; distance traveled, *p* = 0.80; latency, *p* = 0.30) are reported separately with unadjusted *p* values and are not included in the 57 comparisons in this table.

# **5 Supplementary Table S1b. Family 2 — hypothesis-driven follow-up experiments (m = 30)**

| **Experiment** | **Measure** | **Figure** | **n (WT/KO)** | **Raw p** | **q** | **q < 0.05** |
| --- | --- | --- | --- | --- | --- | --- |
| Home cage, MK-801 | Activity after injection | 10d | 20/20 | 0.2445 | 0.324 |  |
| Home cage, methamphetamine | Activity after injection | 10a | 21/21 | 0.2583 | 0.324 |  |
| Microdialysis | Dopamine, AUC | 4i, j | 9/8 | 0.1982 | 0.285 |  |
|  | Dopamine, time course | 4i, j | 9/8 | 0.1043 | 0.196 |  |
| Open field, methamphetamine | Center time | 4e–h | 15/15 | 0.8223 | 0.851 |  |
|  | Stereotypic counts | 4e–h | 15/15 | 0.0165 | 0.049 | **YES** |
|  | Total distance traveled | 4e–h | 15/15 | 0.0128 | 0.044 | **YES** |
|  | Vertical activity | 4e–h | 15/15 | 0.0067 | 0.044 | **YES** |
| Open field, saline | Center time | 4e–h | 15/15 | 0.5138 | 0.558 |  |
|  | Stereotypic counts | 4e–h | 15/15 | 0.3580 | 0.413 |  |
|  | Total distance traveled | 4e–h | 15/15 | 0.1335 | 0.236 |  |
|  | Vertical activity | 4e–h | 15/15 | 0.9567 | 0.957 |  |
| Startle / PPI, clozapine 3 mg/kg | PPI, 110-dB startle | 5j–l | 15/15 | 0.5211 | 0.558 |  |
|  | PPI, 120-dB startle | 5j–l | 15/15 | 0.0240 | 0.060 |  |
|  | Startle amplitude | 5j–l | 15/15 | 0.0094 | 0.044 | **YES** |
| Startle / PPI, haloperidol 0.3 mg/kg | PPI, 110-dB startle | 5d–f | 15/15 | 0.0554 | 0.111 |  |
|  | PPI, 120-dB startle | 5d–f | 15/15 | 0.0020 | 0.030 | **YES** |
|  | Startle amplitude | 5d–f | 15/15 | 0.1521 | 0.254 |  |
| Startle / PPI, haloperidol 1 mg/kg | PPI, 110-dB startle | 5g–i | 15/15 | 0.0106 | 0.044 | **YES** |
|  | PPI, 120-dB startle | 5g–i | 15/15 | 0.0085 | 0.044 | **YES** |
|  | Startle amplitude | 5g–i | 15/15 | 0.2844 | 0.341 |  |
| Startle / PPI, saline (clozapine session) | PPI, 110-dB startle | — | 15/15 | 0.2595 | 0.324 |  |
|  | PPI, 120-dB startle | — | 15/15 | 0.0475 | 0.102 |  |
|  | Startle amplitude | — | 15/15 | 0.0240 | 0.060 |  |
| Startle / PPI, saline (haloperidol 0.3 session) | PPI, 110-dB startle | — | 15/15 | 0.1690 | 0.267 |  |
|  | PPI, 120-dB startle | — | 15/15 | 0.0132 | 0.044 | **YES** |
|  | Startle amplitude | — | 15/15 | 0.0087 | 0.044 | **YES** |
| Startle / PPI, saline (haloperidol 1 session) | PPI, 110-dB startle | — | 15/15 | 0.1998 | 0.285 |  |
|  | PPI, 120-dB startle | — | 15/15 | 0.0012 | 0.030 | **YES** |
|  | Startle amplitude | — | 15/15 | 0.0313 | 0.072 |  |

The 3 antipsychotic sessions were conducted in the same cohort, in the order haloperidol 0.3 mg/kg, clozapine 3 mg/kg, and haloperidol 1 mg/kg. Each session contained four parallel genotype × treatment groups (*n* = 15 per group), and treatment assignments changed across sessions. The genotype effects listed here are within-treatment comparisons; the corresponding drug × genotype interactions are reported separately in Section 8a and Supplementary Table S2. Entries without a figure reference are reported in Supplementary Table S1 only.

# **6 Supplementary Table S1c. Family 3 — gait analysis (m = 171): measures reaching *q* < 0.05**

| **Cohort / condition** | **Measure** | **Raw p** | **q** |
| --- | --- | --- | --- |
| Forelimb, cohort 2 | %PropelStride | 0.00073 | 0.015 |
|  | %BrakeStance | 0.00220 | 0.029 |
|  | %PropelStance | 0.00220 | 0.029 |
|  | Brake | 0.00256 | 0.031 |
| Forelimb, cohort 3, 20 cm/s | %PropelStance | 0.00035 | 0.010 |
|  | %BrakeStance | 0.00035 | 0.010 |
|  | %BrakeStride | 0.00036 | 0.010 |
|  | %PropelStride | 0.00089 | 0.015 |
|  | Propel | 0.00146 | 0.023 |
|  | Brake | 0.00330 | 0.038 |
| Forelimb, cohort 3, 30 cm/s | %PropelStride | 0.00014 | 0.010 |
|  | Absolute PawAngle | 0.00020 | 0.010 |
|  | %BrakeStance | 0.00041 | 0.010 |
|  | %PropelStance | 0.00041 | 0.010 |
|  | Brake | 0.00083 | 0.015 |
|  | %BrakeStride | 0.00354 | 0.038 |

Sixteen of the 171 gait comparisons were significant after correction within the gait family, all of them forelimb comparisons; no hindlimb comparison reached *q* < 0.05. These sixteen entries are not sixteen independent abnormalities. %BrakeStance and %PropelStance partition the stance phase and therefore sum to 100 by definition, which is why they return identical *p* and *q* values within a condition; the absolute durations (Brake, Propel) and the stride-normalized percentages (%BrakeStride, %PropelStride) are further transformations of the same component durations; and the same underlying variables are counted again in each cohort and at each belt speed. The set should therefore be read as convergent evidence for a single change in the timing of forepaw contact, together with one entry for forepaw orientation (absolute paw angle, at 30 cm/s). That no hindlimb comparison reached the threshold is not by itself a test of whether the two limbs were affected differently; that comparison is reported in Section 6a below. The complete list of all 171 comparisons, with group means, effect estimates, 95% confidence intervals, and raw and adjusted *p* values, is provided as Supplementary Data 1; the per-animal source data are provided as Supplementary Data 2.

6a Genotype × limb interaction

Because the forelimb and hindlimb measures were recorded from the same animals in the same run, the two limb pairs are paired within each mouse. For each animal we therefore computed the difference score *D* = forelimb value − hindlimb value and compared *D* between genotypes by a two-tailed *t*-test. This is equivalent to the genotype × limb interaction term of a mixed-model analysis of variance with limb as the within-subject factor, and it is the test of whether the genotype effect differs between the limbs. It was applied to the two measures that index distinct aspects of the phenotype: %BrakeStance for the timing of forepaw contact, and absolute paw angle for paw orientation. %PropelStride is reported for completeness, but it is a further normalization of the same component durations as %BrakeStance and is not an independent test.

Interaction *p* values, %BrakeStance: 0.0002 (cohort 2), 0.0007 (cohort 3, 20 cm/s) and 0.0061 (cohort 3, 30 cm/s). Absolute paw angle: 0.0217, 0.0092 and 0.0003. %PropelStride: 0.0001, 0.0040 and 0.0189. Because %BrakeStance is a bounded percentage and absolute paw angle is a strictly positive quantity, the interaction was repeated on the logit and the log scale respectively; all six interactions remained significant (%BrakeStance, logit: 0.0001, 0.0012 and 0.0202; absolute paw angle, log: 0.0098, 0.0258 and 0.0002). In each of the six conditions the 95% confidence interval of the genotype effect excluded zero for the forelimb and included zero for the hindlimb. Full estimates, confidence intervals and sensitivity analyses are given in Supplementary Data 1, sheet S2_genotype_x_limb.

Stride scaling. Neither stride length nor stride frequency differed between genotypes in the forelimb in any condition (stride length, *p* = 0.26, 0.53 and 0.25; stride frequency, *p* = 0.26, 0.61 and 0.34), and the 95% confidence interval of the genotype difference included zero in every case. The forelimb contact-timing phenotype was therefore not accompanied by a detectable genotype difference in forelimb stride length or stride frequency.

# **7 Replication across cohorts**

Independent-cohort replication provides evidence of reproducibility beyond the multiplicity-adjusted result in a single cohort. We report both the within-family FDR results and replication across independent cohorts. Repeated sessions within the same cohort provide evidence of within-cohort consistency but are not independent replications.

| **Measure** | **Cohort / session** | **n (WT/KO)** | **Raw p** |
| --- | --- | --- | --- |
| Total distance, open field | 2nd cohort (Fig. 2) | 30/29 | 0.0023 |
|  | 1st cohort | 21/21 | 0.0007 |
| Startle amplitude | 2nd cohort (Fig. 5a) | 16/16 | 0.0004 |
|  | 1st cohort | 21/21 | 0.0278 |
| PPI, 120-dB startle | 2nd cohort, drug free (Fig. 5c) | 16/16 | 0.0186 |
|  | 1st cohort, drug free | 21/21 | 0.0035 |
|  | 2nd cohort, saline (session 1) | 15/15 | 0.0132 |
|  | 2nd cohort, saline (clozapine session) | 15/15 | 0.0475 |
|  | 2nd cohort, saline (session 2) | 15/15 | 0.0012 |

The reduction in prepulse inhibition at the 120-dB startle condition was observed in 5 drug-free or saline-treated sessions across 2 cohorts, with a consistent direction and effect size (Cohen's *d* between 0.76 and 1.32). In the primary cohort, *p* = 0.0186 and *q* = 0.212; the reduction was also observed in an independent drug-free cohort (*p* = 0.0035). These results support a reproducible PPI phenotype despite the primary-cohort result not surviving within-family FDR correction. The 3 saline sessions in the primary cohort provide further evidence of consistency within that cohort.

# **8 Drug × genotype interaction in the methamphetamine experiment**

The methamphetamine experiment included a saline arm (*n* = 15 per genotype) run in parallel. The drug × genotype interaction tests below evaluate whether the genotype difference depends on methamphetamine treatment. The unadjusted interaction *p* values for total distance, vertical activity and stereotypic counts provide exploratory evidence of different treatment responses by genotype. A significant comparison in one treatment arm and a nonsignificant comparison in the other would not, by itself, establish an interaction.

| **Measure (post-injection)** | **Saline arm** | **Methamphetamine arm** | **Drug × genotype** |
| --- | --- | --- | --- |
| Total distance traveled | p = 0.134 | p = 0.013 | p = 0.016 |
| Vertical activity | p = 0.957 | p = 0.007 | p = 0.008 |
| Stereotypic counts | p = 0.358 | p = 0.017 | p = 0.029 |
| Time in center area | p = 0.514 | p = 0.822 | p = 0.952 |

**8a Exploratory antipsychotic drug × genotype interactions**

**Design and analysis**

Each antipsychotic session contained WT–saline, KO–saline, WT–drug and KO–drug groups (*n* = 15 per group). The same 60 mice were tested in the order haloperidol 0.3 mg/kg, clozapine 3 mg/kg, and haloperidol 1 mg/kg, with one treatment per mouse per session and changed treatment assignments across sessions. The dose mapping follows the README of Supplementary Data 2: t02, haloperidol 0.3 mg/kg; t03, clozapine 3 mg/kg; t04, haloperidol 1 mg/kg. The t04 acquisition-field label is explicitly documented as incorrect in that README.

The 3 sessions were analyzed separately. Startle amplitude was averaged over the 110- and 120-dB stimuli for each mouse. PPI was averaged over the 74- and 78-dB prepulses separately for each startle intensity. For each outcome, a two-way ANOVA included genotype, treatment and their interaction. Each animal contributed one mean response to an analysis, and all 60 animals contributed to each session analysis. No observations were excluded. Analyses used Python 3.12 with NumPy 2.3.5 and SciPy 1.17.0. Per-animal source data are provided in Supplementary Data 2.

The interaction estimate is (KO − WT)drug − (KO − WT)saline. Pointwise 95% confidence intervals use the pooled residual variance across the four groups and 56 residual degrees of freedom. The 9 interaction *p* values were adjusted together by the Benjamini–Hochberg procedure as a separate retrospective exploratory family. This addition does not alter the original 57-, 30- and 171-test families. As a sensitivity analysis, experiment date was added as a blocking factor within each session. Observations from repeated sessions were not pooled as independent animals.

**Results and interpretation**

No drug × genotype interaction was significant before or after adjustment (unadjusted *p* = 0.198–0.956; *q* = 0.768–0.956). Date-adjusted interaction *p* values were also nonsignificant (*p* = 0.193–0.956). At 120 dB, the changes in the KO-minus-WT PPI difference were −8.99 percentage points for haloperidol 0.3 mg/kg, +6.49 points for haloperidol 1 mg/kg, and −0.65 points for clozapine 3 mg/kg. The confidence intervals span both directions of change. These results do not establish equivalent drug responses, exclude partial improvement or demonstrate pharmacological non-responsiveness. Fixed session order and prior treatment histories also limit interpretation. Specifically, all mice in the clozapine-session saline groups had received haloperidol 0.3 mg/kg in the preceding session, whereas all mice in the clozapine groups had received saline; current treatment was therefore confounded with previous treatment.

**Supplementary Table S2 Exploratory antipsychotic interactions**

| **Drug** | **Outcome** | **Estimate** | **95% CI** | **F** | **p** | **q** |
| --- | --- | --- | --- | --- | --- | --- |
| Haloperidol 0.3 mg/kg | Startle amplitude | -0.27 | -1.02 to 0.49 | 0.500 | 0.482 | 0.768 |
| Haloperidol 0.3 mg/kg | PPI 110 dB | -4.73 | -27.12 to 17.67 | 0.179 | 0.674 | 0.768 |
| Haloperidol 0.3 mg/kg | PPI 120 dB | -8.99 | -31.82 to 13.83 | 0.623 | 0.433 | 0.768 |
| Haloperidol 1 mg/kg | Startle amplitude | -0.18 | -0.88 to 0.52 | 0.268 | 0.606 | 0.768 |
| Haloperidol 1 mg/kg | PPI 110 dB | -6.93 | -40.73 to 26.87 | 0.169 | 0.683 | 0.768 |
| Haloperidol 1 mg/kg | PPI 120 dB | 6.49 | -12.33 to 25.32 | 0.477 | 0.493 | 0.768 |
| Clozapine 3 mg/kg | Startle amplitude | -0.14 | -0.77 to 0.48 | 0.213 | 0.646 | 0.768 |
| Clozapine 3 mg/kg | PPI 110 dB | 21.52 | -11.59 to 54.62 | 1.695 | 0.198 | 0.768 |
| Clozapine 3 mg/kg | PPI 120 dB | -0.65 | -24.16 to 22.86 | 0.003 | 0.956 | 0.956 |

The interaction estimate is (KO − WT)drug − (KO − WT)saline. All F tests have 1 and 56 degrees of freedom. Estimates are in percentage points for PPI and in the source amplitude units for startle. Confidence intervals are pointwise and are not multiplicity-adjusted. *p* is unadjusted; *q* is adjusted across these nine exploratory interactions. Positive PPI estimates indicate an upward shift in the KO-minus-WT contrast relative to saline; they do not by themselves demonstrate normalization.

**Group means for PPI at 120 dB**

| **Drug session** | **WT saline** | **KO saline** | **WT drug** | **KO drug** |
| --- | --- | --- | --- | --- |
| Haloperidol 0.3 mg/kg | 34.14 | 14.11 | 42.54 | 13.52 |
| Haloperidol 1 mg/kg | 41.81 | 17.06 | 47.94 | 29.68 |
| Clozapine 3 mg/kg | 31.64 | 13.56 | 47.98 | 29.25 |

Values are mean PPI (%), averaged over the 2 prepulse levels; *n* = 15 per cell. Per-animal source data are provided in Supplementary Data 2. The gait interaction sheet named S2_genotype_x_limb in Supplementary Data 1 is a separate dataset.

# **9 Code**

The following code reproduces the adjusted *p* values from a table of raw *p* values and family assignments.

## **R**

```
d <- read.csv("pvalues.csv")   # columns: measure, family, p
d$q <- NA
for (f in unique(d$family)) {
  i <- which(d$family == f & !is.na(d$p))
  d$q[i] <- p.adjust(d$p[i], method = "BH")
}
d$within_family_fdr_significant <- d$q < 0.05
write.csv(d, "pvalues_adjusted.csv", row.names = FALSE)
```

## **Python**

```
import pandas as pd
from statsmodels.stats.multitest import multipletests

d = pd.read_csv("pvalues.csv")            # columns: measure, family, p
d["q"] = float("nan")
for f, g in d.dropna(subset=["p"]).groupby("family"):
    d.loc[g.index, "q"] = multipletests(g["p"], method="fdr_bh")[1]
d["within_family_fdr_significant"] = d["q"] < 0.05
d.to_csv("pvalues_adjusted.csv", index=False)
```

# **10 Limitations of this correction**

- FDR adjustment is performed separately within each specified family. A measure significant after correction within Family 2 has been adjusted together with 29 other measures. The 9 added antipsychotic interaction tests have a separate exploratory adjustment; they are not included in the original 30-test family.
- The assignment of measures to families was made after the experiments were performed and was not preregistered.
- The size of Family 1 depends on which measures are reported. Adding or removing measures can change adjusted *p* values through both the family size and the rank ordering of *p* values, and findings whose adjusted values lie close to 0.05 can change status as a result. This applies in particular to the freezing subtraction index for the context test (*q* = 0.058).
- Repeated-measures analyses contribute one *p* value for the genotype main effect. Serial observations within an animal or cage are dependent and should not be treated as independent experimental units. Other scientifically justified definitions of the testing family may yield different adjusted *p* values.
- FDR correction addresses the expected proportion of false discoveries among rejected hypotheses under its statistical assumptions; it does not establish that a finding is reproducible. Conversely, a nonsignificant result does not establish equivalence or absence of a biologically meaningful effect. This applies in particular to nominal findings such as baseline-subtracted contextual freezing (*p* = 0.0041, *q* = 0.058) and PPI at 120 dB after clozapine (*p* = 0.0240, *q* = 0.060), which remain candidates for further confirmation.
